# Robust linear DNA degradation supports replication–initiation-defective mutants in *Escherichia coli*

**DOI:** 10.1093/g3journal/jkac228

**Published:** 2022-09-27

**Authors:** T V Pritha Rao, Andrei Kuzminov

**Affiliations:** Department of Microbiology, University of Illinois at Urbana-Champaign, Urbana, IL 61801, USA; Department of Microbiology, University of Illinois at Urbana-Champaign, Urbana, IL 61801, USA

**Keywords:** synthetic lethality, *oriC*, DnaA, DnaC, DnaN, RecA, RecBCD, RuvA, RecG, Rep, recombinational repair, linear DNA degradation, chromosomal DNA synthesis, replication initiation, replication elongation

## Abstract

RecBCD helicase/nuclease supports replication fork progress via recombinational repair or linear DNA degradation, explaining *recBC* mutant synthetic lethality with replication elongation defects. Since replication initiation defects leave chromosomes without replication forks, these should be insensitive to the *recBCD* status. Surprisingly, we found that both *Escherichia coli dnaA46*(Ts) and *dnaC2*(Ts) initiation mutants at semi-permissive temperatures are also *recBC*-colethal. Interestingly, *dnaA46 recBC* lethality suppressors suggest underinitiation as the problem, while *dnaC2 recBC* suppressors signal overintiation. Using genetic and physical approaches, we studied the *dnaA46 recBC* synthetic lethality, for the possibility that RecBCD participates in replication initiation. Overproduced DnaA46 mutant protein interferes with growth of *dnaA*^+^ cells, while the residual viability of the *dnaA46 recBC* mutant depends on the auxiliary replicative helicase Rep, suggesting replication fork inhibition by the DnaA46 mutant protein. The *dnaA46* mutant depends on linear DNA degradation by RecBCD, rather than on recombinational repair. At the same time, the *dnaA46* defect also interacts with Holliday junction-moving defects, suggesting reversal of inhibited forks. However, in contrast to all known *recBC*-colethals, which fragment their chromosomes, the *dnaA46 recBC* mutant develops no chromosome fragmentation, indicating that its inhibited replication forks are stable. Physical measurements confirm replication inhibition in the *dnaA46* mutant shifted to semi-permissive temperatures, both at the level of elongation and initiation, while RecBCD gradually restores elongation and then initiation. We propose that RecBCD-catalyzed resetting of inhibited replication forks allows replication to displace the “sticky” DnaA46(Ts) protein from the chromosomal DNA, mustering enough DnaA for new initiations.

## Introduction

**Fig. 1.**
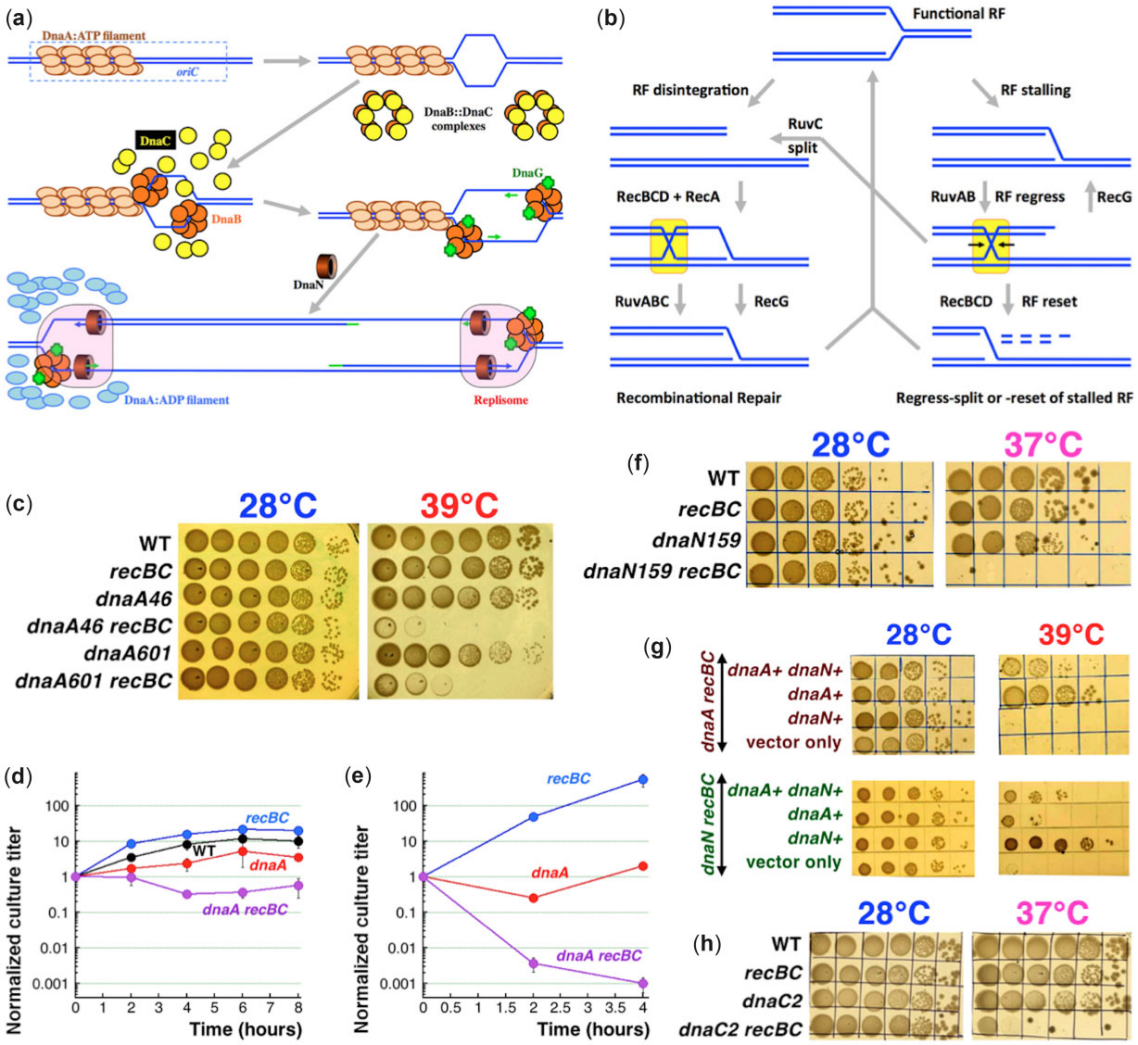
The phenomenon of the *dna*(Ts) *recBC*(Ts) synthetic lethality. a) A scheme of the replication initiation in *E. coli*. b) Two major processes produce HJs in DNA: recombinational repair is on the left, RF regress/reversal is on the right. HJs are highlighted. c) Here and on, serial 10-fold dilutions of growing cultures were spotted in rows, from undiluted culture to 10^−5^ dilutions, and developed at the indicated temperatures. A minimum of 2 independent spottings was done for every mutant combination. Two different *dnaA* alleles were used here, *dnaA46* and *dnaA601*. Subsequently, only *dnaA46* allele was used (referred to simply as *dnaA*), unless indicated otherwise. Similarly, since mostly the *recBC*(Ts) allele was used (unless specified otherwise), we referred to it as “*recBC*.” Throughout the paper, WT = AB1157. d) Titer of the indicated mutants in liquid culture, when grown at 28°C to OD_600_ = 0.1, then shifted to 39°C. Here and on, all values are means of 3 or more independent measurements ± SEM (that is, when error bars are almost touching, the 2 values are NOT different). When no error bars are visible, they are covered by the symbols. e) Titer of the indicated mutants, when grown at 28°C to OD_600_ = 0.1, diluted ×30 and then shifted to 39°C, all in liquid culture. f) Colethality of the *dnaN159*(Ts) *recBC* double mutant at 37°C. Henceforth, *dnaN159*(Ts) is referred to as *dnaN*. g) Complementation with plasmids, harboring the indicated genes (*dnaA+*, or *dnaN+*, or both), to test if the *dnaA recBC* colethality is due to a cryptic *dnaN* defect. Only the 5 serial dilutions are shown. h) Colethality of the *dnaC2*(Ts) *recBC* double mutant at 37°C. Henceforth, *dnaC2*(Ts) allele is referred to as *dnaC*.

Chromosomal replication in *Escherichia coli* begins at *oriC*, when, as detected in vitro, the DnaA protein in complex with ATP binds to specific short sequences in the origin DNA called DnaA boxes (Fuller and Kornberg 1983; Fuller *et al.* 1984; Sekimizu *et al.* 1987). The *oriC* DNA has several high- and low-affinity DnaA boxes, promoting limited DnaA polymerization into a spiral multisubunit DnaA-ATP filament (Fig. 1a), with the *oriC* DNA wrapped along the outside of the filament (Fuller *et al.* 1984; Erzberger *et al.* 2006; Miller *et al.* 2009). The resulting positive supercoiling in the *oriC* DNA is proposed to cause compensatory hyper-negative supercoiling within the nearby AT-rich DNA sequence, promoting its unwinding into a single-strand bubble (Erzberger *et al.* 2006) (Fig. 1a). In the next step, as observed in vitro, DnaC loads 2 DnaB helicase hexamers at the opposite corners of the single-strand bubble (Funnell *et al.* 1987; Marszalek and Kaguni 1994), followed by recruitment of DnaG primase and, after the synthesized primers are loaded with the DnaN clamps, of the replisome itself, that begins bidirectional DNA synthesis (Fang *et al.* 1999) (Fig. 1a). Mapping of the priming sites around *oriC* shows, however, that the initial replication is unidirectional, going away from the DnaA-bound cluster, while the other direction is activated later and away from *oriC*, both in vivo (Kohara *et al.* 1985) and in vitro (Fang *et al.* 1999). Most likely, the second fork is activated, as the first Okazaki fragment on the lagging strand of the “first fork” becomes its leading strand. The progress of the fork in the opposite direction displaces the DnaA filament off *oriC* DNA (Fig. 1a), using the DnaN-riding Hda protein (as explained later).

Apart from *oriC*, high-affinity DnaA boxes are also found at the *datA* locus, that converts initiation-competent ATP-DnaA into initiation-inactive ADP-DnaA and thus regulates initiation by controlling the levels of ATP-DnaA (Kitagawa *et al.* 1998; Kasho and Katayama 2013). DnaA is also known to regulate transcription of several genes, including itself (Atlung *et al.* 1985; Braun *et al.* 1985; Kücherer *et al.* 1986) and, generally, binds to more than 300 sites all over the chromosome (Hansen and Atlung 2018).

Chromosomal replication in bacteria is extremely fast and accurate under optimal conditions, but since only 2 replication forks (RFs) traverse the entire chromosome per cell cycle, it takes *E. coli* a minimum of 40 min to finish chromosome duplication (Helmstetter 1987). Part of the reason replication cannot be faster is that replisomes are slowed down by encounters with DNA damage, heavily transcribed genes or tightly bound proteins, which cause RF stalling and even disintegration (Bierne and Michel 1994; Kuzminov 1995a, 1995b; Cox *et al.* 2000) (Fig. 1b). Disintegrated RFs are reassembled by recombinational repair enzymes RecBCD, RecA and RuvABC/RecG (Kuzminov 1999); DNA synthesis at a restored or abandoned fork structure resumes after replisome reloading by PriA (Michel and Sandler 2017). For example, the *rep* mutant in *E. coli*, famous for its slow RFs (Lane and Denhardt 1975), is susceptible to fork stalling and disintegration (Michel *et al.* 1997). This makes the *rep* mutant synthetic lethal with the *recBC* defect (Uzest *et al.* 1995), meaning that the RecBCD function is essential for the *rep* mutant survival. In general, synthetic lethality (or “colethality”—a simpler term which we use sometime) is inviability of a combination of 2 viable mutants (Guarente 1993; Nijman 2011).

The RecBCD enzyme is a helicase-nuclease that vigorously degrades linear DNA, but having recognized a Chi-site, generates 3' overhangs at double strand DNA ends (Dillingham and Kowalczykowski 2008), which are then used by the RecA protein for homology-guided strand invasion into an intact duplex DNA (Cox 1993; Kuzminov 1999) (Fig. 1b, the left pathway). The invasion creates a Holliday junction (HJ) intermediate, which is resolved by either the RuvABC resolvasome or by the RecG duplex DNA pump (Fig. 1b, the left pathway) (Kuzminov 1999). In the absence of RecBCD, the stalled forks in the *rep* mutants undergo RuvAB-dependent RF reversal forming an HJ, which is eventually cleaved by RuvC (Seigneur *et al.* 1998) (Fig. 1b, the right column). A similar stalling, reversal and cleavage of the fork in the other replichore causes release of linear sister chromosome arm (detectable as chromosome fragmentation in pulsed-field gels) and the *rep recBC* synthetic lethality (Uzest *et al.* 1995; Michel *et al.* 1997). If RecBCD is present, however, it degrades the extruded DNA from the reversed fork, preventing such double strand breaks by RuvC and allowing the fork to reset (Seigneur *et al.* 1998) (Fig. 1b, the bottom right).

Apart from *rep*, various *E. coli* replication elongation mutants, that experience fork problems, either in the replicative helicase DnaB, or in the β-clamp, α, or ψ subunits of the replicative polymerase, have been shown to experience RuvABC-dependent double strand breaks as a result of fork reversal and to require RecBCD for viability (Michel *et al.* 1997; Flores *et al.* 2001; Grompone *et al.* 2002). At the same time, mutants impaired for replication initiation functions, *dnaA* and *dnaC* (Fig. 1a), were assumed to be indifferent toward recombinational repair status of the cell, as without initiation there are no RFs in the chromosome that may require repair. True, DnaC also functions away from *oriC*, when it helps restart the disintegrated forks reassembled by recombinational repair (Michel and Sandler 2017), but this DnaC function is expected to idle in the *recBCD* mutants, where disintegrated forks are not repaired.

Therefore, we were surprised to find that a combination of defective DnaA and RecBCD proteins is also colethal in *E. coli* (Fig. 1, c and e). Initially, we reasoned that either the *dnaA*(Ts) mutant suffers from (unexpected) double strand breaks which require RecBCD for repair or, more interestingly, the *recBC* mutant is mechanistically compromised for replication initiation, further exacerbating the *dnaA* defect. To distinguish between the 2 scenarios, we have carried out genetic and physical assays. Perhaps not surprisingly, neither of our original ideas were supported by our findings, as the primary problem appears to be the tighter binding of the mutant DnaA protein to the chromosomal DNA, which blocks RF progress. Linear DNA degradation by RecBCD alleviates this block, while the resulting steady RF progress releases the DNA-bound mutant DnaA protein off the chromosome, making it available for initiation at the origin.

## Materials and methods

### Bacterial strains, growth conditions, and chemical reagents

*Escherichia coli* strains used in this study are all K-12 derived and are described in Supplementary Table 1. The strains were grown in LB (per 1 l: 10 g of tryptone, 5 g of yeast extract, 5 g of NaCl, pH to 7.4 with 250 µl of 4 M NaOH; LB agar contained 15 g of agar/l of LB broth) at 28°C unless mentioned otherwise.

### Plasmids and cloning

Plasmids are described in Supplementary Table 2. Plasmids pNRK416 and pMob-dnaKJ were provided by Benedicte Michel (CNRS, France). For the construction of pPR1 (*dnaA+ dnaN+*) plasmid, genomic DNA of MG1655 and pMTL20 were both digested with NcoI and ligated to create a plasmid library. The plasmid library was transformed into DH5α *dnaA46* strain and plated at 42°C to select for suppressors of temperature sensitivity of DnaA46. One clone containing insert was selected and called pPR1. pPR1 was digested with XhoI and religated to create the *dnaA+* (*dnaN*–) plasmid pPR7. pPR1 was digested with NcoI, religated and transformed into DH5α *dnaA46* to create a plasmid with insert in the opposite orientation. Plasmid pPR2 was isolated from suppressors that grew and checked for inversion of orientation. pPR2 was digested with SphI and religated to create the *dnaN+* (*dnaA*–) plasmid pPR8.

#### Cloning *dnaA* and *dnaA46* genes in pMTL20

Primers GCTATTCCATGGTACGGGCTGATG and GCTATCAAGCTTGTGCCACCATTTCCATCTCG were used to amplify the *dnaA*+ and *dnaA46* genes. Upon amplification the insert and vector were digested with NcoI and HindIII, ligated and transformed into DH5α *dnaA46* to select for suppressors. Plasmid DNA was isolated from the suppressors, and inserts were verified by sequencing.

#### Cloning of *hslUV*+ into pMTL20

Primers CTCTCGGAATTCGCAGCTGGTTGAAGTTCCGT and CTGCCCATGGGATGAAAATGATTGAACGCG were used to amplify the wild type *hslUV* genes. The amplicon and the plasmid pMTL20 were digested with EcoRI and NcoI and ligated.

### Spot test

Overnight cultures were diluted 100-fold to subculture and then grown at 28°C to OD_600_ = 0.1–0.15. Strains containing plasmids were subcultured in the presence of antibiotic to maintain the plasmid. Serial 10-fold dilutions were made in 1% NaCl, and 10 µl of the original culture, as well as all dilutions up to 10^−5^ were spotted on LB plates and incubated at the indicated temperatures for 24–48 h (to yield colonies of similar sizes).

### Viability assay

Overnight cultures were diluted 100-fold and grown at 28°C to OD_600_ = 0.1–0.15. The cultures were shifted to 39°C either directly or were further diluted 30-fold, shaken at 28°C for 20 min to acclimatize to the diluted conditions and moved to 39°C. At appropriate times, aliquots were removed, serially diluted, and spotted on LB plates. Colonies were counted while still small under stereomicroscope, after 18–24 h of incubation of plates at room temperature.

*Insertional mutagenesis* with pRL27 (Larsen *et al.* 2002) to isolate suppressors followed our established protocols (Rotman *et al.* 2009; Budke and Kuzminov 2010).

### Origin and terminus kinetics

Overnight cultures were subcultured 100-fold and grown at 28°C to OD_600_ = 0.1–0.15, when the cultures were further diluted 30-fold, shaken at 28°C for 20 min to acclimate to the diluted conditions and moved to 39°C. Twenty milliliters of *recBC dnaA* culture, 10 ml of *dnaA*, *recBC dnaA seqA*::pRL27 and *recBC dnaA hslUV*::pRL27, and 10, 8, 3, 3, and 3 ml of *recBC* cultures were taken at, correspondingly, 0, 1, 2, 3, and 4 h after shift to 39°C to isolate DNA in duplicate agarose plugs. The cells in plugs were lysed for 16 h at 65°C in the lysis buffer (see the next section for its composition). After lysis, the plugs were washed with 1 ml of TE for 10 min thrice, in 1 ml of 0.25 N HCl for 20 min, in 1 ml 0.5 M NaOH for 20 min and in 1 M Tris–HCl for 20 min. The DNA from plugs was transferred using vacuum transfer and hybridized with either the probe for the origin or the terminus, which were described before (Kouzminova and Kuzminov 2012), for 24 h in hybridization buffer (5% SDS, 0.5 Na_2_HPO_4_, pH 7.4), then washed with 0.1× hybridization buffer and exposed to a phosphorimager screen.

### Quantification of chromosome fragmentation

Overnight cultures were subcultured 100-fold and grown at 28°C to OD_600_ = 0.1–0.15 in the presence of ^32^P label, as described (Khan and Kuzminov 2012), then half of the cultures was moved to either 37°C or 39°C. Depending on the culture density, 1–0.25 ml aliquots were removed after 4 h of shaking, and cells were collected by centrifugation. Cell pellets were resuspended in 60 µl TE (10 mM Tris–HCl, 1 mM EDTA, pH 8.0), mixed with 5 µl of 5 mg/ml Proteinase K (Roche Applied Science, final concentration in plugs is 200 µg/ml) and 65 µl of molten agarose in lysis buffer (1.2% agarose in 1% laurylsarcosine, 50 mM Tris–HCl, 25 mM EDTA, pH 8.0) and poured into plug molds (Bio-Rad). The plugs were lysed for 16 h at 65°C in the lysis buffer. Half-plugs were inserted into the wells of 1% agarose in 0.5× Tris–borate–EDTA buffer. The gel was run at 12°C in Bio-Rad CHEF-DR II pulsed field gel electrophoresis system, at 6 V/cm, with initial switch time 60 s and the final switch time 120 s, for 20 h. The gel was then dried and exposed to a phosphorimager screen. The exposed screen was analyzed with the FLA-3000 fluorescent image analyzer (FujiFilm). The data were analyzed using Image Gauge version 3.41 software (FujiFilm). The percentage of chromosomal fragmentation was calculated as signal in the lane below the well divided by the total signal in the lane + well and multiplied by 100.

### Rate of DNA synthesis

Overnight cultures were subcultured 100-fold and grown at 28°C to OD_600_ = 0.1–0.15, 200 µl aliquots were taken at each time point and mixed with 1 µCi of ^3^H-dT, and the reaction was incubated at 39°C for 2 min, after which 5 ml of ice-cold 5% TCA was added to stop further incorporation. The TCA precipitate was collected on filters (Whatman, grade GF/C) and washed with 5 ml of 5% TCA, followed by 5 ml of ethanol. One hundred microliters of 100 mM KOH was spotted on the filter and dried, to quench fluorescence. The filters were incubated with scintillation fluid overnight before counting; ^3^H counts of the filters were measured in Beckman-Coulter LS 6500 scintillation counter.

### Detection of branched DNA structures

Overnight cultures were subcultured 100-fold and grown at 28°C to OD_600_ = 0.1–0.15, when the cultures were diluted 30-fold, shaken at 28°C for 20 min to acclimate to the dilution and moved to 39°C for 4 h. DNA was isolated from 25 ml of *dnaA*, 50 ml of *dnaA recBC*, and from 1 ml of *recBC* strain by phenol–chloroform extraction. Briefly, cell pellet was suspended in 50 µl of 30% sucrose in TE and lysed with 350 µl of 2% SDS in TE after mixing by inversion and 5 min at 65°C. The lysate was extracted first with 400 µl of phenol, then with 200 µl of phenol/200 µl of chloroform mixture and, lastly, with 400 µl of chloroform. The aqueous phase was transferred to a fresh tube, and, after adding 20 µl of 5 M NaCl and 1 ml of ethanol, DNA was precipitated by multiple tube inversions. The pellet was dissolved in 500 µl TE, and DNA was reprecipitated using NaCl and ethanol. Four hundred nanograms of DNA for each strain was digested with XmnI or StuI + NdeI, and the reaction was run in 0.7% agarose on 1× Tris–acetate–EDTA buffer. The gel was treated with 0.25 N HCl for 20 min, 0.5 M NaOH for 20 min, and 1 M Tris–HCl pH = 8.0, for 20 min, DNA was transferred to nylon membrane by capillary transfer, and the membrane was hybridized with *oriC-*specific probe.

## Results

### The phenomenon of RecBCD-dependent replication initiation

#### Colethality of *dnaA recBC*

As mentioned in the *Introduction*, synthetic lethality is regularly observed when recombinational repair defects are combined with replication elongation defects (Michel *et al.* 1997; Flores *et al.* 2001; Grompone *et al.* 2002). Here, we unexpectedly found colethality between defects in recombinational repair and replication *initiation*. The *dnaA46* allele is progressively defective in binding ATP at higher temperatures (Carr and Kaguni 1996), so it no longer supports cell growth above 40°C due to a block in replication initiation (Hirota *et al.* 1970), but it still forms (smaller) colonies at 39°C, its semipermissive temperature (Fig. 1c). The *recB270 recC271* (*recBC*(Ts)) mutant is disabled for both DNA degradation and repair at 37°C and above (Kushner 1974; Kouzminova and Kuzminov 2004), but still spots essentially like WT at 39°C (Fig. 1c). At the same time, the *dnaA46 recBC*(Ts) double mutant struggles to grow at 39°C (all strains show normal growth at 28°C) (Fig. 1c)—making 39°C nonpermissive for the double mutant. Another temperature-sensitive allele, *dnaA601*, that carries the same mutation as *dnaA46* in the ATP-binding site (Hansen *et al.* 1992), shows a similar colethality with *recBC*(Ts) (Fig. 1c). Unless otherwise mentioned, we have used the *dnaA46* and *recBC*(Ts) alleles for the rest of our study; henceforth, we will refer to these 2 mutations simply as *dnaA* or *recBC*.

To see whether the inability of the *dnaA recBC* mutant to form colonies at the nonpermissive temperature reflects stasis or actual death, we shifted the 4 strains to 39°C while still in the liquid cultures and continued incubation for 8 more hours. As expected, the WT and *recBC* mutant strains grew to saturation, while the titer of the *dnaA* mutant increased slowly (Fig. 1d), consistent with lighter colonies in the plating assay (Fig. 1c). The titer of the *dnaA recBC* mutant was mostly static (Fig. 1d), suggesting that 39°C was inhibitory for the double mutant, rather than lethal. However, diluting the cultures 30 times or deeper before shifting them to 39°C, while still supporting viability of the single *dnaA* mutant, does lead to fast and dramatic loss of titer in the double mutant cultures (Fig. 1e). This low-cell-density-dependent colethality of the *dnaA recBC* double mutant explains why we always observed residual growth in the highest density spot of the strain (Fig. 1c). Our subsequent physical analyses have 2 conditions for *dnaA recBC* mutant: the undiluted static one (Fig. 1d) vs the diluted lethal one (Fig. 1e). Physical assays with diluted cultures, although of higher distinguishing power, were less practical because they required significant culture volumes.

#### Other synthetic lethal combinations

The *dnaN* gene, which encodes the DNA clamp that makes the DNA Pol III processive (Fig. 1a), is immediately downstream of *dnaA* in the same operon (Sakakibara *et al.* 1981). The *dnaN159*(Ts) defect (henceforth *dnaN*) leads to RF stalling at nonpermissive temperatures and, as many other elongation defects, is colethal in combination with the *recBC* defect at its semipermissive temperature of 37°C (Fig. 1f) (Grompone *et al.* 2002). Since DnaA autoregulates itself transcriptionally by binding to the DnaA box at the *dnaA* promoter (Atlung *et al.* 1985; Braun *et al.* 1985; Kücherer *et al.* 1986), the observed *dnaA recBC* colethality could have been due to *dnaN* expression problems instead. We tested this possibility by complementing the *dnaA recBC* strain with the functional *dnaA* or *dnaN* genes. We found that, while *dnaA*-only expressing plasmid could rescue the *dnaA recBC* colethality, the *dnaN*-only expressing plasmid failed to do so (Fig. 1g). At the same time, we confirmed that the *dnaN recBC* colethality is due to a defective *dnaN* gene, as it was complemented by the *dnaN*-expressing plasmid, but not by the *dnaA*-expressing plasmid (Fig. 1g). Thus, the *dnaA recBC* colethality is due to the *dnaA* defect, rather than a cryptic *dnaN* defect.

Besides its main DNA clamp function, DnaN also plays a role in modulating initiation at *oriC*, as the DnaN–Hda protein complex stimulates ATP–DnaA disassembly from the origin DNA via ATP hydrolysis (Fig. 1a) (Kato and Katayama 2001). Thus, the *dnaN recBC* strain could be lethal due to a defect in initiation regulation (we will test this possibility later). DnaC is the DnaB inhibitor-chaperone and the second *E. coli* function specific for replication initiation (Fig. 1a) (Wickner and Hurwitz 1975; Wahle *et al.* 1989). Two types of *dnaC*(Ts) mutants are known, all completely defective at 42°C: *dnaC1* and *dnaC7* block replication elongation (Carl 1970; Wechsler 1975), while *dnaC2* and *dnaC28* block replication initiation (Carl 1970; Schubach *et al.* 1973). To explore the possibility that inactivation of any initiation function makes *recBCD* mutants inviable, we tested the viability of the *dnaC2*(Ts) *recBC* mutant. Unexpectedly, we found that the *dnaC2*(Ts) defect (henceforth *dnaC*) is also synthetic lethal with the *recBC* defect at the semipermissive temperature of 37°C (Fig. 1h). Thus, various problems with chromosomal replication initiation lead to RecBCD-dependence.

#### Suppressors of the *dnaA recBC* colethality

To understand the nature of colethality in the *dnaA recBC*, *dnaN recBC*, and *dnaC recBC* double mutants, we isolated suppressors for each of these combinations after insertional mutagenesis. Suppressors were isolated as colonies growing at the nonpermissive temperature for the given colethal (39°C for *dnaA recBC*, 37°C for either *dnaN recBC* or *dnaC recBC*) and, before identification, were confirmed to still suppress after reintroduction into the original colethal mutant. If the 3 colethalities had a common nature, they would have been suppressed by inactivation of the same or similar functions.

**Fig. 2.**
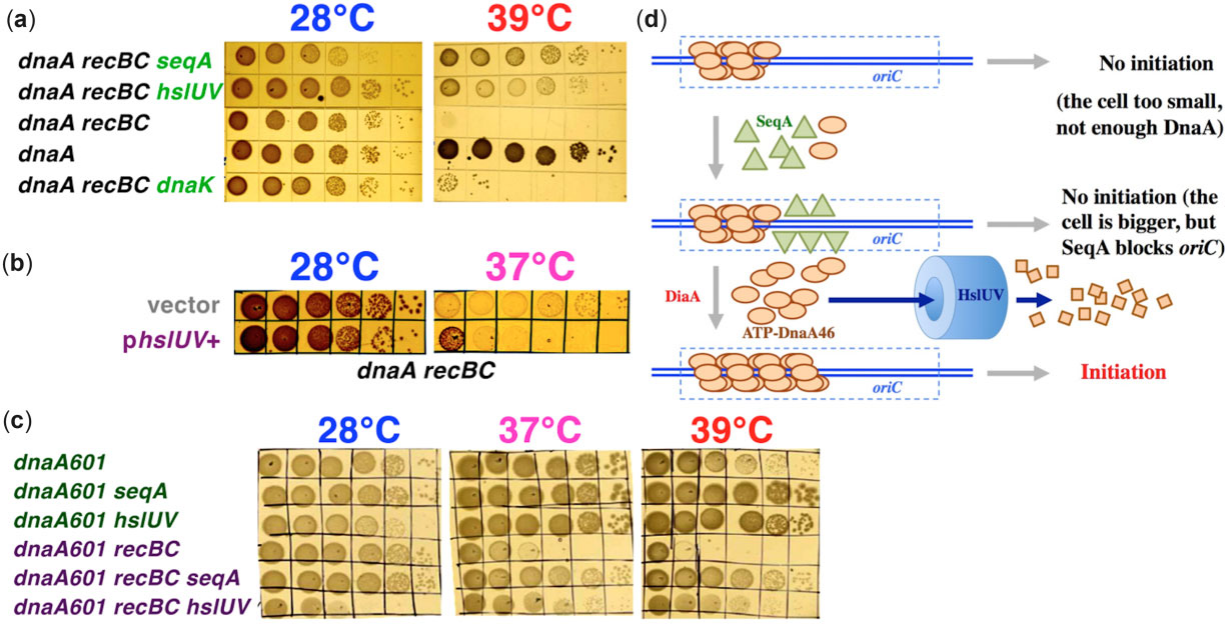
Suppressors of the *dnaA recBC* colethality. a) Suppressor phenotypes at 28°C and 39°C. b) Overexperssion of HslUV genes exacerbates the *dnaA recBC* growth at 37°C. c) Both the *seqA* and the *hslUV* suppressors also suppress the *dnaA601 recBC* colethality at 37°C and 39°C. d) A possible explanation of how inactivation of SeqA and HslUV could suppress the *dnaA46* initiation defect.

The *dnaA recBC* colethality was suppressed by insertions at only 2 loci (Fig. 2a;Supplementary Fig. 1, a and b). We found 18 independent insertions all over the *hslUV* operon encoding one of the 5 major ATP-dependent proteases of *E. coli* and a homolog of the eukaryotic 26S proteasome (Coux *et al.* 1996). An *hslUV* over-expressing plasmid exaggerates the *dnaA recBC* colethality at 37°C (Fig. 2b), confirming suppression by *hslUV* inactivation. HslUV protease is proposed to degrade the unstable DnaA46 protein at higher temperatures (Katayama *et al.* 1996), offering the most likely mechanism of *hslUV* suppression (Fig. 2d).

We also isolated 4 insertions in and upstream of *seqA* gene as suppressors of the *dnaA recBC* colethality (Fig. 2a;Supplementary Fig. 1, a and b). The *seqA* suppressors were visibly stronger than the *hslUV* ones (Fig. 2, a and c). One of the *seqA* suppressors was tried and found to also suppress the colethality in the *dnaA recBC* strain that carries the *hslUV*-overexpressing plasmid (data not shown). The SeqA protein organizes *E. coli* chromosome in 2 important ways. SeqA forms filaments to organize the sister-chromatid cohesion zone behind the RF (Waldminghaus *et al.* 2012; Helgesen *et al.* 2015) via binding to hemi-methylated GATC sites (Brendler *et al.* 2000; Guarné *et al.* 2005). Equally important is SeqA binding at the hemi-methylated *oriC*, that negatively regulates replication initiation (Lu *et al.* 1994; von Freiesleben *et al.* 1994) by blocking long DnaA filament formation until *oriC* is fully methylated (Fig. 2d), thus setting the eclipse period, the minimal time between successive initiations (von Freiesleben *et al.* 2000; Olsson *et al.* 2002). This SeqA binding to the origin likely interferes with initiations by the partially active DnaA46 protein at higher temperatures; without SeqA, *oriC* will be available for DnaA polymerization any time (Fig. 2d).

We have also found that both the *hslUV* and *seqA* defects similarly suppress the *dnaA601 recBC* colethal combination and improve growth of *dnaA601* at 39°C (Fig. 2c). However, in contrast to the *dnaA46* mutant, the *dnaA601* mutant is inhibited by additional copies of the mutant protein at 28°C (Hansen *et al.* 1992)—providing a likely explanation of the poor growth of the *dnaA601 hslUV* and especially of the *dnaA601 recBC hslUV* strains at this temperature (Fig. 2c). We also isolated a single insert upstream of *dnaK*, but this promising lead failed to confirm (see the Supplementary Results and Supplementary Fig. 1).

#### Suppressors of the *dnaN recBC* colethality

**Fig. 3.**
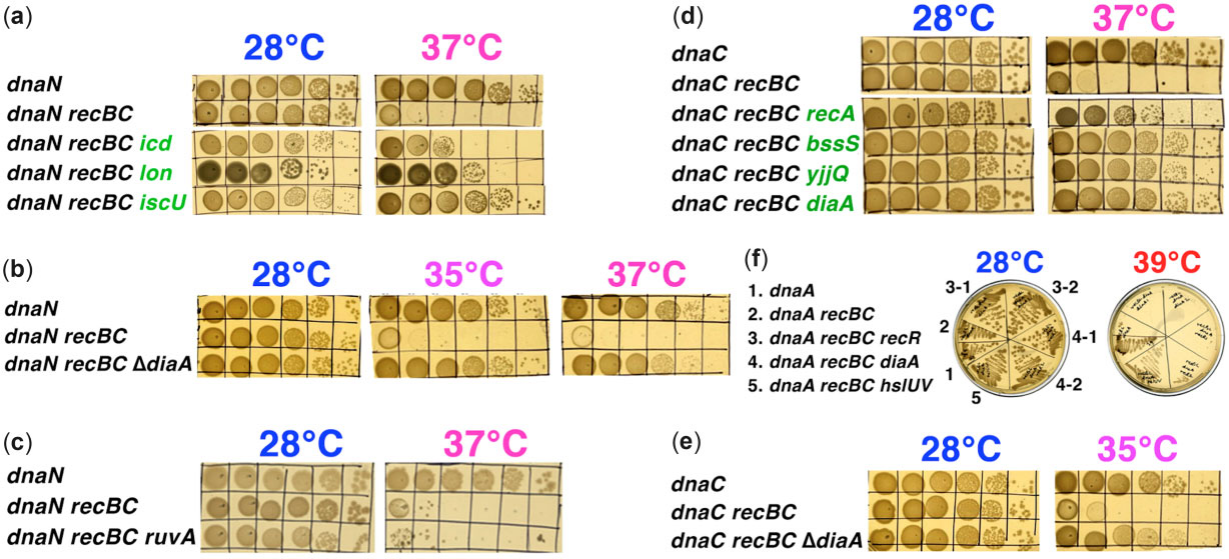
Suppressors of the *dnaN recBC* vs the *dnaC recBC* colethality. a) The *dnaN recBC* suppressor phenotypes at 28°C and 37°C. b) The *dnaN recBC* colethality is suppressed by the Δ*diaA* allele. c) Inactivation of RuvA fails to suppress the *dnaN recBC* colethality. d) The *dnaC recBC* suppressor phenotypes at 28°C and 37°C. e) The *dnaC recBC* colethality is suppressed by the Δ*diaA* allele; suppression is better at 35°C, than at 37°C. f) The *recR* and *diaA* suppressors of *dnaC recBC* fail to suppress *dnaA recBC* colethality. The layout at both temperatures is the same, so only 1 plate is marked; 3-1 and 3-2 are independent transductants of *dnaA recBC recR*; 4-1 and 4-2 are independent transductants of *dnaA recBC diaA*.

The *dnaN recBC* colethality was suppressed by 6 independent insertions in the *iscRSUA* operon (Fig. 3a;Supplementary Fig. 2). The *iscRSUA* operon and the *hscBA* operon downstream are responsible for the synthesis and repair of iron-sulfur clusters that are used by key enzymes of the central metabolism (Vinella *et al.* 2009). We have also obtained insertions in the *hscA* gene which is a chaperone interacting with the IscU protein, as well as in *pta*, *icd*, and *ubiC* genes, all related to the central metabolism. As a result of their metabolic defects, all these suppressors grew slower (Fig. 3a, the 28°C plate).

Similar to isolating *dnaA recBC* suppressors in a major protease, 4 of our *dnaN recBC* suppressors had inserts in and upstream of the *lon* gene (Fig. 3a;Supplementary Fig. 2), which codes for another major ATP-dependent protease (Tsilibaris *et al.* 2006), suggesting that the DnaN(Ts) protein at the semipermissive temperatures is targeted by Lon. There was a single insert in the mismatch repair gene *mutH*, which we did not pursue further (Supplementary Fig. 2). Finally, one more insertion was in the *diaA* gene, coding for a positive initiation factor, the DiaA protein, which assists ATP-DnaA polymerization at *oriC* for replication initiation (Fig. 2d) (Ishida *et al.* 2004). Indeed, a Δ*diaA* allele rescues the *dnaN recBC* double mutant (Fig. 3b), strongly suggesting that the *dnaN recBC* colethality is caused by replication over-initiation.

Michel and colleagues have shown that the *dnaN recBC* double mutant undergoes RuvABC-dependent chromosome fragmentation, proposing this as an explanation for its colethality (Grompone *et al.* 2002). We inactivated the *ruvA* gene to test if blocking chromosome fragmentation suppresses the colethality, but found that the resulting *dnaN recBC ruvA* strain is still lethal at 37°C (Fig. 3c). Thus, prevention of RuvABC-dependent double strand DNA breaks at reversed RFs (Fig. 1b) does not rescue the *dnaN recBC* mutant.

#### Suppressors of the *dnaC recBC* colethality

The *dnaC recBC* colethality was suppressed by 3 inserts into and upstream of *diaA* (Fig. 3d;Supplementary Fig. 3); moreover, *diaA* deletion also rescues the *dnaC recBC* colethality (Fig. 3e). Thus, inactivation of the DnaA polymerization-promoting factor DiaA (Fig. 2d) suppresses both the *dnaC recBC* and *dnaN recBC* colethalities, strongly suggesting that the chromosome problems in both cases are caused by replication over-initiation.

We also found that insertions in both *recR* and *recA* genes suppress the *dnaC recBC* colethality (Fig. 3d;Supplementary Fig. 3). The RecF, RecO, and RecR proteins work together in the RecA-mediated pathway to repair blocked single strand DNA gaps (Kuzminov 1999), and expression of both *recR* and especially *recA* is elevated in the *dnaC* mutant at 38°C (Løbner-Olesen *et al.* 2008). It looks like DnaC malfunction causes formation of blocked single strand gaps that apparently trigger recombinational misrepair or SOS induction by RecFOR—similar to what is proposed to happen during thymineless death (Fonville *et al.* 2010; Kuong and Kuzminov 2010). Alternatively, RecAFOR-initiated repair could also require DnaC-dependent loading of the replisome by PriA—while without replication restart, the unresolved daughter-strand gap repair intermediates generate problems that only RecBCD-catalyzed repair can fix. The suppression by inactivation of the central activity of recombinational repair, the RecA protein, looks counterintuitive, but in fact makes sense, as there are only 2 recombinational repair pathways in *E. coli* cells, RecBCD and RecFOR, and both are already blocked in the suppressed *dnaC recBC recR* triple mutant.

Insertions inactivating the *bssS* gene and between the *yjjP* and *yjjQ* genes also suppressed the *dnaC recBC* colethality (Fig. 3d;Supplementary Fig. 3). The latter insert is only 3 genes away from the *dnaC* itself and therefore could modulate *dnaC* expression. There were some more single suppressing inserts that we did not pursue further (Supplementary Fig. 3). Instead, we introduced the *hslUV* suppressor of the *dnaA recBC* colethality into the *dnaC recBC* double mutant, but it failed to suppress (data not shown). And vice versa, the *diaA* and *recR* suppressors of the *dnaC recBC* colethality failed to suppress the *dnaA recBC* colethality (Fig. 3f), highlighting the fact that even though both DnaA and DnaC are initiation functions, the nature of the *dnaA recBC* colethality is distinct from the *dnaC recBC* one.

Overall, suppressor analysis showed that synthetic lethality of the *recBC* defect with the replication initiation problems could have different nature, although some similarities were observed, too. In particular, suppression by protein degradation defects was expected for Ts-alleles, via stabilization of the mutant proteins at the semipermissive temperatures. Also, since both the *dnaC recBC* and *dnaN recBC* colethalities are suppressed by the *diaA* defect, they must be caused by replication over-initiation, which is a known reason of colethality with the *recBCD* defect in *E. coli* (Grigorian *et al.* 2003; Simmons *et al.* 2004; Rotman *et al.* 2014; Khan *et al.* 2016). In contrast, the *dnaA recBC* colethality is suppressed by the *seqA* defect and therefore must be caused by insufficient initiation (Fig. 2d), raising a possibility of a direct mechanistic RecBCD participation in the process of replication initiation. Thus, for the rest of the paper, we concentrate on the more mysterious *dnaA recBC* colethality, using the *dnaC recBC* and *dnaN recBC* colethalities in some physical assays as reference points.

### Genetics of the *dnaA recBC* colethality

#### Recombinational repair is not required in the *dnaA* mutant

**Fig. 4.**
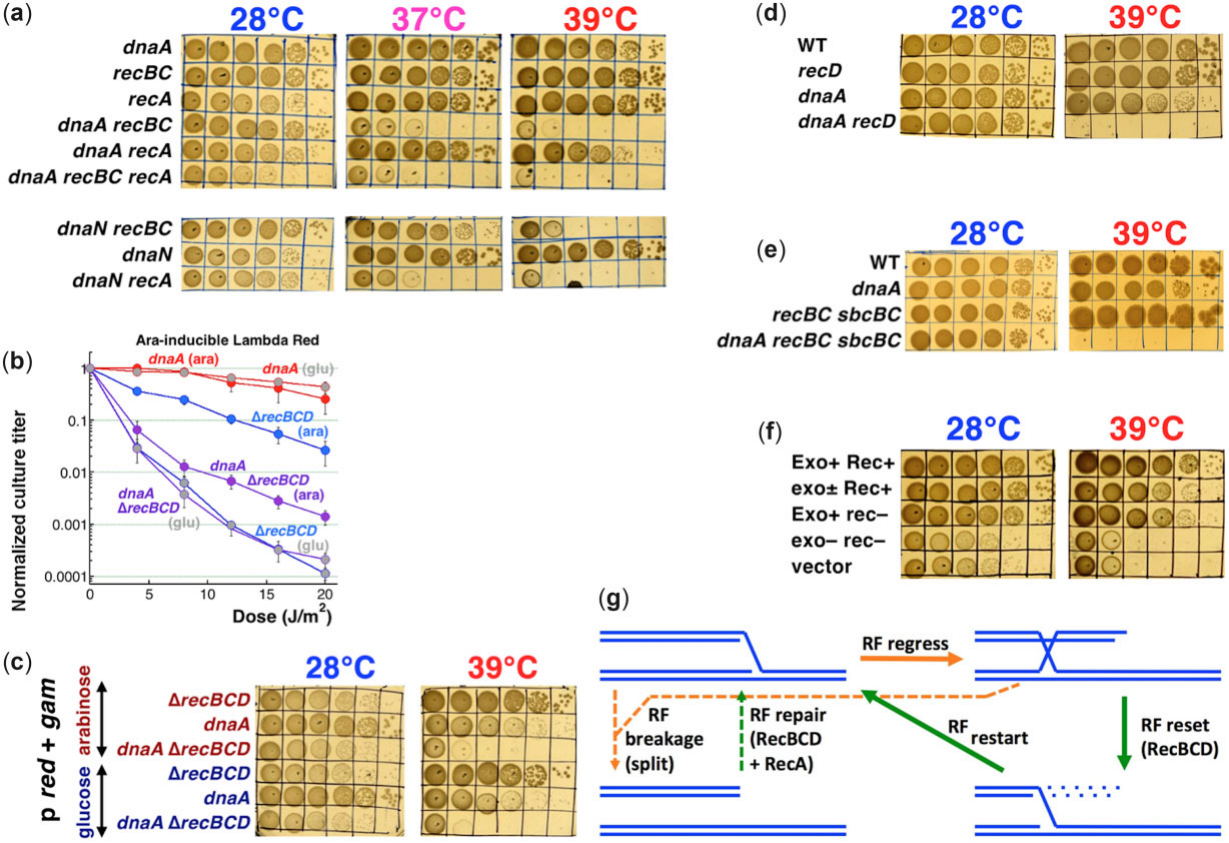
The *dnaA* mutant relies on the linear DNA degradation activity of RecBCD, rather than on its recombinational repair activity. a) The *dnaA recA* combination is not lethal, while *dnaN recA* is. b) Arabinose-induction of lambda-Red recombinational repair system increases UV-survival of the Δ*recBCD* mutants. The strains harbor pKD46 plasmid, which expresses phage lambda *red-Alpha*, *red-Beta* (and *red-Gamma*) genes, restoring recombinational repair in the Δ*recBCD* mutant. Glucose (glu) turns the expression OFF, arabinose (ara) turns it ON. c) Expression of the lambda Red genes (glucose—OFF, arabinose—ON) does not improve *dnaA recBCD* plating at 39°C. d) The *dnaA recD* combination is lethal. e) The *dnaA recBC sbcBC* combination is lethal. f) Rescue of the *dnaA* Δ*recBCD* coinhibition by plasmids expressing various point mutants of the RecBCD enzyme. The alleles and plasmids are: Exo^+^ Rec^+^ (WT), pAMP1; exo^±^ Rec^+^ (Δ*recD*), pAMP3; Exo^+^ rec^–^ (*recB* K1082Q), pAMP5; exo^–^ rec^–^ (*recB* D1080A), pAMP7; vector, pWSK29. g) A scheme of 2 possible ways RecBCD could help with rescue: (1) (dashed lines) in case of RF disintegration, RecBCD helps RecA to reassemble the fork; (2) (solid lines) in case of RF regress, RecBCD degrades the open end to eliminate the HJ, resetting the fork with no help from RecA.

As mentioned in the *Introduction*, the RecBCD enzyme has 2 important roles in the cell: (1) degradation of linear DNA; (2) processing of a double-strand DNA end to prepare the substrate (3'-ss-end) for RecA polymerization, a prerequisite for recombinational repair. To test the role of recombinational repair in the viability of the *dnaA* mutant directly, we constructed the *dnaA recA938* (null) double mutant. We observed that the *recA* defect shows only a weak interaction with the *dnaA* defect, further inhibiting, but not blocking, the growth of the double mutant (Fig. 4a). Likewise, introducing the *recA938* defect in the *recBC dnaA* strain did not exacerbate the colethality beyond the expected growth inhibition (Fig. 4a). As a positive control for *recA-*dependence, we confirmed that the *dnaN recA938* combination is even more colethal than the *dnaN recBC* one (Fig. 4a, bottom). Thus, the viability of the *dnaA recA* double mutant all but rules out that the *dnaA* mutant relies upon the recombinational repair function of RecBCD.

We have further tested the role of recombinational repair, separate from linear DNA degradation, by introducing the plasmid pKD46 (Datsenko and Wanner 2000) into the *dnaA* Δ*recBCD* strain. This plasmid, by supplying phage lambda recombination functions, significantly restores recombinational repair capacity to the Δ*recBCD* mutant (Fig. 4b), without restoring linear DNA degradation capacity. We found that, though the strain *dnaA* Δ*recBCD* pKD46 (+ ara) also shows enhanced UV survival (Fig. 4b) (suggesting partial restoration of recombinational repair), it still cannot grow at 39°C (Fig. 4c). Thus, the dependence of the *dnaA* mutant on RecBCD is not due to recombinational repair, suggesting that it is due to linear DNA degradation. And this switch from recombinational repair to DNA degradation dependence is clearly due to the *dnaA* mutation, as the difference in UV-survival between Δ*recBCD* (ara) and the *dnaA ΔrecBCD* (ara) curves demonstrates.

#### DNA degradation by RecBCD is essential in the *dnaA* mutant

The above reasoning is further supported by the surprisingly strong lethality of the *dnaA recD* combination (Fig. 4d). The *recD* mutant is defective in the linear DNA degradation activity, but is fully recombination-proficient (Amundsen *et al.* 1986)—in fact, it is a hyper-rec in all homologous recombination assays, compared to WT (Chaudhury and Smith 1984; Lovett *et al.* 1988). This strong *dnaA recD* colethality confirms that normal recombinational repair capacity is not important for the *dnaA* mutant, while the normal linear DNA degradation capacity appears to be. The mutant essentially WT for recombinational repair yet completely deficient in linear DNA degradation is *recBC sbcBC* (Lloyd and Buckman 1985; Rao and Kuzminov 2019); in combination with the *dnaA* at 39°C, this mutant shows perhaps the strongest lethality reported in this work (Fig. 4e), suggesting that recombinational repair without linear DNA degradation becomes poisonous for the *dnaA*(Ts) mutant at the semipermissive temperature.

In order to conclusively determine which of the 2 general functions of RecBCD is required for survival of the *dnaA* mutants at 39°C, we introduced RecBCD mutant-carrying plasmids, which would restore either one of the 2 functions of the RecBCD complex—recombinational repair or DNA degradation, in the *dnaA recBC* background (Miranda and Kuzminov 2003). Since the chromosomally expressed RecBC(Ts) protein could interfere with the RecBCD proteins supplied by the plasmids, we did this complementation in the *dnaA* Δ*recBCD* background (Fig. 4f). The low copy number plasmid (∼6 per chromosome) introduces a mild overexpression of various RecBCD variants, which have no effect at 39°C in either Δ*recBCD* or in *dnaA* single mutants (Supplementary Fig. 4). We found that introduction of either recombination function plus partial DNA degradation (Rec^+^ Exo^±^), or full DNA degradation capacity without recombination (Rec^–^ Exo^+^), was sufficient to restore the viability in the *dnaA* Δ*recBCD* strain (Fig. 4f). We conclude that the *dnaA* strain requires linear DNA degradation function of the RecBCD protein for its survival, with no requirement for recombinational repair.

These requirements could be explained if, in the *dnaA* mutants, RFs stall and regress, yet do not break—because regressed RFs can be reset by RecBCD-dependent linear DNA degradation without repair, while broken RFs have to be reassembled by RecA-dependent recombinational repair (Fig. 4g).

#### The *recG* defect suppresses the *dnaA ruvA* coinhibition

**Fig. 5.**
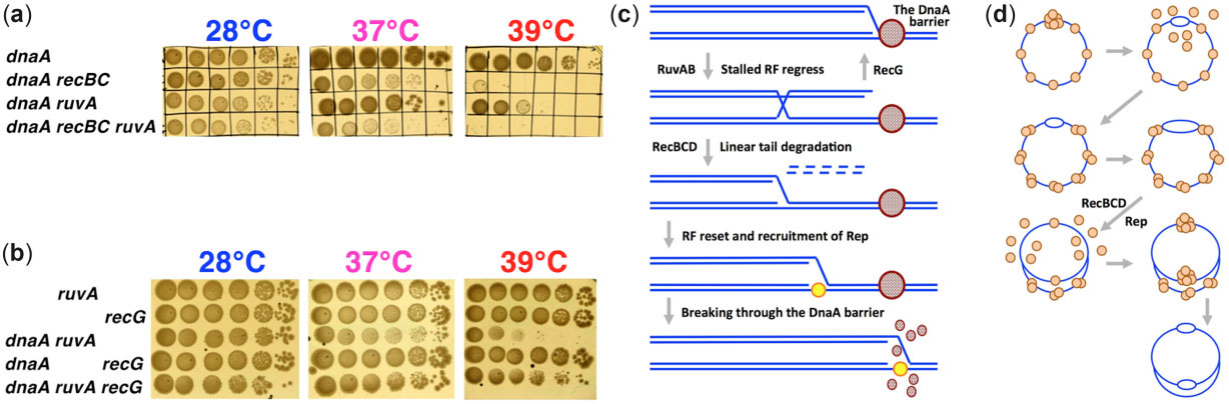
Effects of the HJ migration mutants help to formulate a model of the *dnaA recBC* colethality. a) The *dnaA ruvA* combination is mildly lethal, while addition of *ruvA* to the *dnaA recBC* double mutant exacerbates the defect, rather than suppressing it. b) The *recG* inactivation suppresses the *dnaA ruvA* colethality. c) Our model of the *dnaA recBC* colethality, based on how RecBCD with the help of RuvAB and Rep helicase (the yellow circle) overcomes DnaA RF barrier. RecG is shown to interfere by returning the regressed RF back to its original “stalled” configuration. d) When this scenario plays out at the level of the chromosome replication, not only the RF progress, but also new initiations from the origin will be blocked, if the RecBCD step is not performed.

To further guide our thinking, we introduced defects in HJ migration enzymes into our *dnaA* mutant. HJs, the 4-way fully duplex DNA junctions, form either during recombinational repair or by RF reversal (Fig. 1b, highlighted in yellow). Since the *dnaA* mutants do not depend on RecA, they do not need to resolve HJ after RecA-catalyzed recombintational repair; however, they may still need to move around HJs resulting from RF regress/reversal. Two activities handle HJs in *E. coli* (Kuzminov 1996). The RuvAB DNA-junction-associated pump moves junctions around—so that the RuvC nuclease, that comes later and works at specific resolution sites, could split junctions in half by a pair of symmetrical nicks (Fig. 1b)—but the resulting disintegrated fork would require recombinational repair—which does not happen in our case. The second activity, the RecG dsDNA pump, is known to only push the junctions around. Interestingly, while both activities are required to remove HJs after recombinational repair, they seem to push the junctions in opposite directions (Fig. 5c), judging by their opposite phenotypes in some genetic systems (Foster *et al.* 1996; Harris *et al.* 1996).

It is important to mention that the *recG* defect, although only in combination with the *tus* mutation (inactivates replication termination sites) and a special rpoB* allele (alleviates replication–transcription conflicts), suppresses the *dnaA46* initiation defect even at 42°C (Rudolph *et al.* 2013). Because of this, we did not test the *recG* inactivation in the *dnaA recBC* background, expecting either no effect or maybe a small growth improvement. Inactivation of HJ processing and resolution by the *ruvA* defect in the *dnaA recBC* strain did not rescue the strain (Fig. 5a), as was reported for the *rep recBC* colethals (Seigneur *et al.* 1998). In fact, the *ruvA* defect showed mild colethality in combination with the *dnaA* defect alone (Supplementary Fig. 5, a and b), suggesting chromosomal problems in the *dnaA* mutant that require moving HJs, most likely RF regression. At the same time, the *recG* defect, that inactivates the second DNA junction-pushing activity (Kuzminov 1996), actually suppresses this synthetic inhibition of the *dnaA ruv* mutants (Fig. 5b), as if, after RF stalling, RecG and RuvA do move the resulting HJs in the opposite directions: RuvAB in the “right” direction, while RecG in the “wrong” one. However, *recG* defect (even alone) could ease the *dnaA* defect directly, via alternative initiations (Rudolph *et al.* 2013). At the same time, the lack of improvement of the *dnaA recBC* mutants poor survival at 39°C by inactivation of RuvA (in the *dnaA recBC ruvA* triple mutant) (Fig. 5a) shows that the chromosomal problems in *dnaA recBC* are not the result of HJ resolution by RuvC resolvase (which is made inactive by the *ruvA* defect).

#### Formulating a model

At this point, results of others and our own observations allowed us to formulate a model of chromosomal events in the *dnaA46* mutant, to guide our subsequent inquiry. One requirement to such a model was to explain the importance of linear DNA degradation by RecBCD and of moving around HJs by RuvAB vs RecG—both without subsequent recombinational repair (Fig. 1b or Fig. 4g). The second requirement was to explain how RecBCD-promoted degradation around these HJs facilitates an apparent release of DnaA from the chromosomal DNA to ensure new initiations at the origin, compensating for this mutant’s underinitiation.

We considered 2 specific facts about the *dnaA46* defect: (1) in vitro, the mutant DnaA46 protein binds ∼10 times more readily to *oriC*-containing DNA than WT DnaA protein does, at both low and high temperatures (Carr and Kaguni 1996); (2) due to defective DnaA46 autoregulation at higher temperatures, expression from the *dnaA* promoter region in the *dnaA46* mutant is induced up to 4-fold upon shift from 30°C to 42°C (Atlung *et al.* 1985; Braun *et al.* 1985; Kücherer *et al.* 1986). Therefore, starting with the safe assumption that the mutant DnaA protein binds tighter (than WT DnaA) to its multiple sites on the chromosomal DNA in vivo and therefore stalls RFs at some of them, we propose that RecBCD-promoted linear degradation is needed to restart the stalled forks (Fig. 5c). Thus, the temperature-sensitivity of our *dnaA*(Ts) *recBC*(Ts) strain is not due to a tighter binding of the mutant DnaA protein to its chromosomal sites at 39°C [it binds tighter at any temperature (Carr and Kaguni 1996)], but due to 2 factors at higher temperatures: (1) the deficiency in linear DNA degradation of our *recBC*(Ts) mutant; (2) the increased expression of the auto-regulated DnaA(Ts) protein. One obvious prediction from the idea—since the *dnaA46* mutant at any temperature depends on reset of stalled RFs, it should be synthetic lethal, even at 30°C, with defects in RF restart—was already tested: the *dnaA46 priC* mutant is indeed synthetic lethal at 30°C (Hinds and Sandler 2004).

Specifics of the model include explanation of the *ruvA* and *recG* effects, as well as how recombinational repair is avoided. Since in the stalled forks the replisomes are still intact, such stalled forks will be hard to reverse and so need RuvAB’s help to do so, while RecG would catalyze reverse branch migration (Whitby *et al.* 1993), converting the resulting HJs back to RFs, which is counter-productive (Fig. 5c). We further postulate that, even though the RFs are eventually reversed, they are not split by RuvC resolution, because such splitting would make it all dependent on recombinational repair (Fig. 1b or Fig. 4g), which we do not observe. The independence of recombinational repair of the *dnaA* mutant also suggests that the generated linear tails at reversed forks are relatively short (much shorter than ∼5 kb) and therefore do not have a chance to initiate repair at Chi-sites, as the longer tails would—otherwise recombinational repair would have had an effect. Chi-sites are 8 bp-long special sequences, found in the *E. coli* chromosomal DNA once per ∼5 kb, at which progressing RecBCD switches from the DNA degradation mode to the DNA repair mode (Myers and Stahl 1994). The short linear tail is simply degraded by RecBCD, which resets the fork (Fig. 5c). The fork resetting event likely recruits the Rep helicase as the booster motor for the replicative helicase DnaB (Guy *et al.* 2009; Atkinson, Gupta, and McGlynn 2011), which allows the “turbo” fork to disperse the DnaA barrier, then Rep dissociates. In the cell, Rep associates with the replisomes only transiently, but at any given moment colocalizes with 70% of RFs (Syeda *et al.* 2019). Overall, the action of RecBCD and Rep makes RFs more processive.

Thus, according to the model, RecBCD does not release DnaA from the chromosome directly by degrading long linear DNA tails; rather, steady DnaA release from the chromosomal DNA is promoted by processive replication, supported by RecBCD and Rep. Mechanistically, the DnaA release by progressing RFs in *E. coli* is likely the function of the Hda protein, which tightly associates with the DNA clamp within active replisomes (Kawakami *et al.* 2006). Furthermore, we imagine that, at the chromosomal level, the increased processivity of RFs allows DnaA redistribution from the chromosomal binding sites to the *oriC* polymerization site (Fig. 5d). The interesting corollary of such thinking is that not only replication elongation is expected to be inhibited in the *dnaA recBC* mutants, but also new initiations would be blocked, due to the general unavailability of DnaA (Fig. 5d).

The model generates other testable predictions: (1) any manipulation with the copy number of the major ATP-DnaA hydrolysis site *datA* should exacerbate *dnaA recBC* colethality (as explained below); (2) as a tighter DNA binder, the DnaA46 mutant protein, especially if overproduced, should inhibit growth of *dnaA^+^* cells; (3) the auxiliary helicase Rep should be required for survival in the *dnaA* mutants; (4) chromosomal fragmentation should be minimal in the *dnaA recBC* mutant—mostly suggested by lack of need for recombinational repair; (5) in the *dnaA* (RecBCD^+^) cells at 39°C, the overall replication will be inefficient, due to the complicated mode of conflict resolution, while in the *dnaA recBC* mutants at the 39°C, the replication will be severely inhibited, both at the level of elongation *and* initiation. Below we tested these predictions.

### Testing the model of the *dnaA recBC* colethality

#### Effect of the *datA* deletion or multiple copies of *datA*

The copy number of DnaA is constant and is about 1,000–2,000 copies per rapidly growing cell (Sekimizu *et al.* 1988), distributed in normally growing cells between ∼20% ATP-DnaA (efficient DNA binding) and ∼80% ADP-DnaA (inefficient DNA binding) (Kurokawa *et al.* 1999). Chromosome distribution of the DNA-bound DnaA is dynamic and maintained at its optimum by the ATP-DnaA to ADP-DnaA ratio. There are 2 processes (due to complex names known by their acronyms) that convert ATP-DNA to ADP-DnaA, thus promoting DnaA redistribution: RF (Hda)-dependent “RIDA,” happening at the origin and throughout the chromosome (Kato and Katayama 2001), and (IHF)-dependent “DDAH,” happening continuously at the *datA* chromosomal locus (Kasho and Katayama 2013). There are also 2 processes that continuously recharge DnaA with ATP: one operates at 2 DnaA-reactivation sequences in the chromosome (Fujimitsu *et al.* 2009), while the other uses membrane phospholipids (Garner and Crooke 1996).

**Fig. 6.**
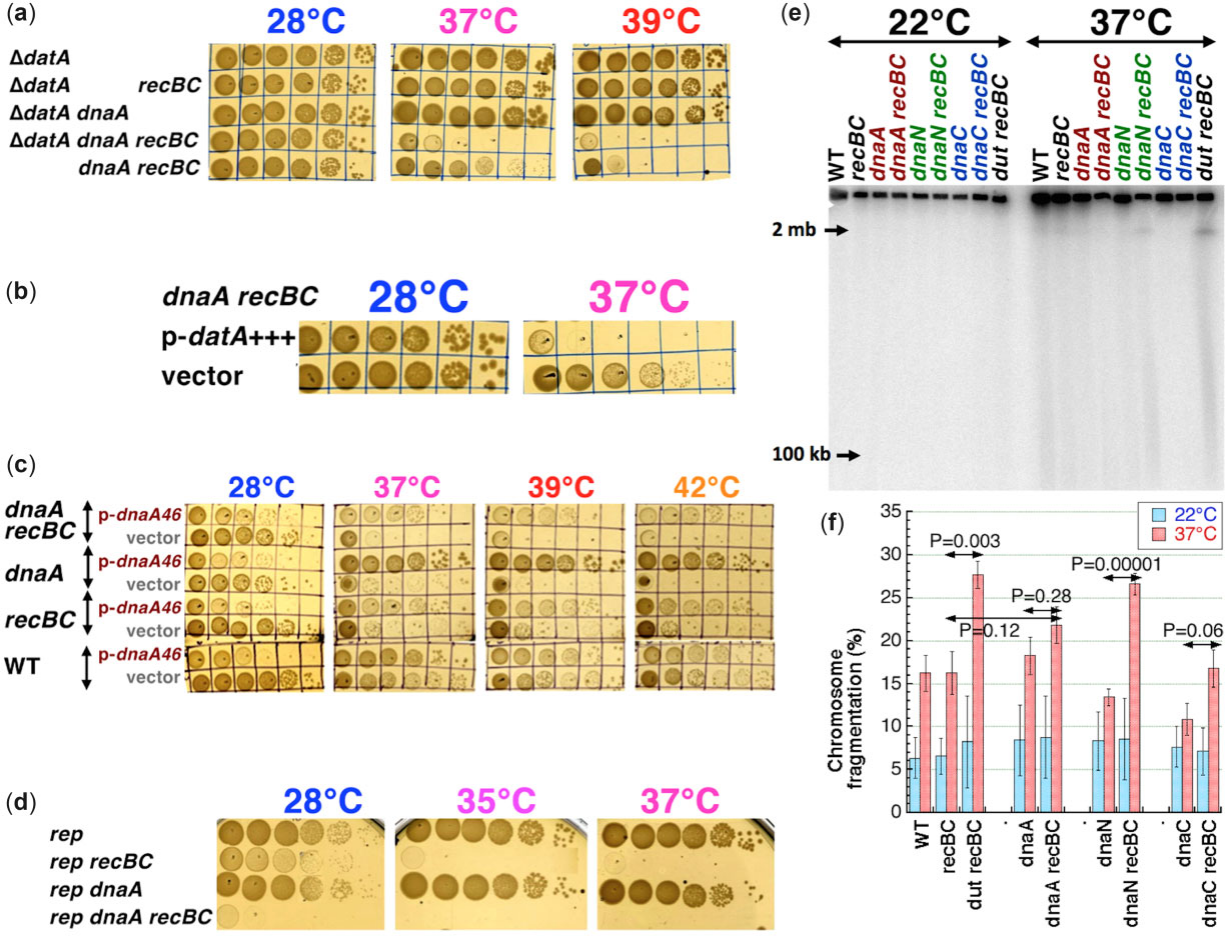
Testing the model. a) The effect of the *datA* deletion on the *dnaA recBC* colethality. b) The effect of several copies of *datA* on the *dnaA recBC* colethality. Vector is pACYC177, the p-datA^+++^ plasmid is pMOR6. c) The effect of high copy number DnaA46 protein in the indicated strains. Vector is pMTL20. d) The effect of the *rep* deletion on the *dnaA recBC* colethality. e) A representative pulsed-field gel showing chromosome fragmentation in various mutants, grown either at 22°C or 37°C for 4 h. Intact chromosomal DNA stays in the wells, subchromosomal fragments smear into the gel. f) Quantification of chromosomal fragmentation from several gels like in “e”. Data are averages ±SEM. Significance of the differences between the corresponding single and double mutants at 37°C is indicated.

If the main problem of the *dnaA recBC* colethal is inadequate accumulation of the mutant DnaA protein at the origin due to the tight DnaA46 binding to its chromosomal sites (in other words, RIDA is underperforming in the *dnaA46* mutant), this problem will be exacerbated by upsetting the ATP-DnaA to ADP-DnaA ratio in either direction. Inactivation of DDAH by deleting *datA* should further increase the ATP-DnaA fraction, thus slowing down the ATP hydrolysis-stimulated redistribution among its chromosomal sites. This is expected to further exacerbate the problem of the mutant with a tighter-DNA-binding DnaA, which, as we postulate, already suffers from the inefficient redistribution of DnaA—and indeed it does (Fig. 6a). Paradoxically, increasing the copy number of *datA* sites in the cell, by bringing in a *datA*-carrying multicopy plasmid, should decrease the ATP-DnaA/ADP-DnaA ratio, making initiation in general less efficient—which should also exacerbate the *dnaA recBC* defect, and it again does (Fig. 6b). Thus, either decreasing the DnaA chromosome redistribution rate by increasing the fraction of ATP-DnaA, or decreasing initiation by decreasing the fraction of ATP-DnaA, both exacerbate the *dnaA recBC* colethality—and both are consistent with the primary defect being tighter mutant DnaA binding to its multiple chromosomal sites.

#### Overexpressed DnaA46 protein inhibits *dnaA*+ cells

According to our model, overproduction of the DnaA46 mutant protein should interfere with growth of *dnaA*^+^ cells. To test this prediction, we cloned the WT *dnaA* and *dnaA46* genes in pMTL20 (Chambers *et al.* 1988) and introduced these constructs in AB1157, *recBC*, *dnaA*, and *dnaA recBC* strains (Fig. 6c;Supplementary Fig. 5). Overexpression of the DnaA46 protein was toxic for all strains at 28°C, this toxicity being somewhat relieved at higher temperatures of 37, 39, and 42°C, especially in the *dnaA* single mutant (Fig. 6c). The inhibitory effect of overproduction of DnaA46 protein and similar DnaA(Ts) mutant proteins at lower temperatures in *dnaA*+ cells was noted before (Hansen *et al.* 1992; Carr and Kaguni 1996; Nyborg *et al.* 2000). High copy number of DnaA46 was able to completely rescue the matching *dnaA* mutant at the higher temperatures, while allowing the double *dnaA recBC* mutant to grow (slowly) at all these temperatures (Fig. 6c). In other words, increasing the concentration of defective DnaA46 compensates for the defect in its function at higher temperatures—consistent with HslUV protease inactivation suppressing the *dnaA recBC* colethality (Fig. 2). Overproduction of several DnaA(Ts) proteins compensates for the temperature-sensitivity of the correspondent mutants (Hansen *et al.* 1992).

Interpretation of these results is somewhat complicated by the fact that our high-copy number vector (pMTL20) itself interfered with growth of the *dnaA*+ strains at these higher temperatures, while the same plasmid, but carrying *dnaA46* gene, improved the growth at these temperatures (Fig. 6c). Perhaps this effect is due to the presence of a single DnaA binding site on pMTL plasmids, but we have not tested this idea. This vector interference with growth at 42°C could be again observed with strains carrying WT *dnaA* constructs, but in this case, overexpressing WT DnaA was only mildly inhibitory at 28°C and allowed all 4 strains to plate at full titer at 42°C (Supplementary Fig. 5).

#### The *dnaA recBC* requires Rep even at 28°C

As explained in the introduction, the overall RF movement is slower in the *rep* mutants due to frequent pauses (Lane and Denhardt 1975; Guy *et al.* 2009; Atkinson, Gupta, Rudolph, *et al.* 2011), while in vitro the Rep helicase boosts the DnaB motor to plough RFs through obstacles in the template DNA (Guy *et al.* 2009; Atkinson, Gupta, and McGlynn 2011). If the DnaA46 protein clusters bound to DNA indeed represent a hindrance to RFs, the activity of the Rep helicase should become essential in the *dnaA recBC* mutant at lower temperatures. We have found that at 28°C, the *rep dnaArecBC* triple mutant is severely inhibited, compared to the *rep dnaA* or *rep recBC* double mutants, and is completely dead at 35°C, suggesting increased impediments ahead of forks (Fig. 6d). The *rep recBC* double mutant is itself synthetic lethal at 37°C [nonpermissive for *recBC*(Ts)] (Uzest *et al.* 1995) and is inhibited for growth even at lower temperatures (Fig. 6d), demonstrating that the weakened activity of mutant RecBCD compromises survival of cells that cannot power-up their RFs. This is also substantiated by the more severe inhibition of the *rep* mutant by overexpression of DnaA46, compared with WT DnaA at 28°C (Supplementary Fig. 6). Expectedly, the DnaA46-carrying plasmid is tolerated better by this strain at higher temperatures, while the WT DnaA-carrying plasmid shows no temperature effect (Supplementary Fig. 6).

#### The *dnaA* defect does not cause chromosome fragmentation

A colethality of a particular mutation with the *recBC* defect usually indicates formation of double-strand breaks as a result of interference with DNA replication (Kuzminov 1999; Michel *et al.* 2001). The 2 major endogenous reasons for RF instability causing double-strand breaks are (1) increased spontaneous DNA damage and intermediates of its excision repair; (2) general inhibition of RF progress (Fig. 1b). It is the second reason that could potentially result in chromosomal fragmentation in the *dnaA* mutants. A useful characteristic of the *recBC* mutants is quantitative “preservation” of their levels of chromosomal fragmentation—because these mutants neither repair double-strand breaks, nor degrade linear DNA (Michel *et al.* 1997). We compared the 3 synthetic lethal strains: *dnaA recBC,dnaN recBC*, and *dnaC recBC* at the non-permissive temperatures for their *recBC*(Ts) alleles for their levels of chromosomal fragmentation (Fig. 6, e and f).

For practical reason, at this point we had to switch from plating, convenient for viability studies, but taking days to develop—to liquid cultures, which facilitate physical studies over shorter time spans. For example, chromosome fragmentation is typically quantified after 4 h at the nonpermissive temperature (Kouzminova *et al.* 2004). We found that *dnaN recBC* strain shows ∼16% chromosomal fragmentation above the background at 37°C (Fig. 6, e and f), but little fragmentation at 39°C [data not shown, was also reported before (Grompone *et al.* 2002)], while the *dnaC recBC* strain shows ∼5% chromosome fragmentation over the background at 37°C (Fig. 6, e and f) and similar numbers at 39°C (data not shown)—which would likely become significant with more repetitions. At the same time, we could not detect any statistically significant fragmentation in the *dnaA recBC* strain over the *dnaA* single or *recBC* single mutant background, at either 37°C (Fig. 6, e and f) or 39°C (data not shown), indicating that the *dnaA* defect does not result in appreciable number of breaks at both RFs at the same time. The undetectable chromosome fragmentation in the *dnaA* mutant is a deviation from a typical behavior of RecBCD-dependent mutants observed so far, but is predicted by our model of the *dnaA recBC* defect (Fig. 5c) and is consistent with its complete independence of recombinational repair (Fig. 4).

#### Both replication initiation and existing fork elongation are blocked in the *dnaA recBC* double mutant

**Fig. 7.**
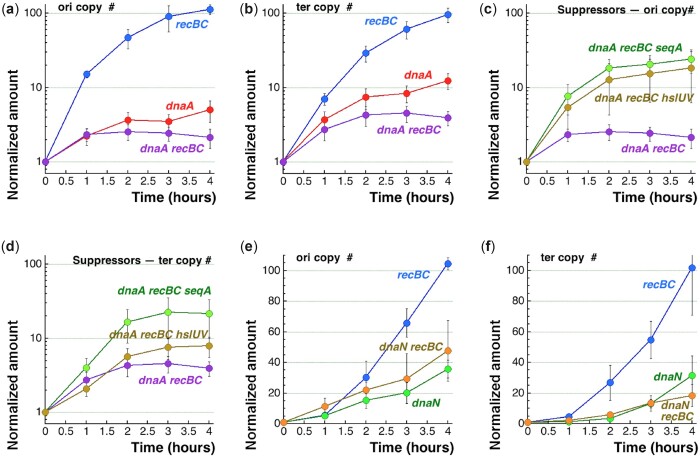
Chromosome replication in the *dnaA recBC* vs *dnaN recBC* mutants at the semi-permissive temperatures is followed by increase of the ori or ter copy number. The temperature is 39°C when the *dnaA* mutants are involved, 37°C when the *dnaN* mutants are involved. a, c, e) Increase of the origin copy number in the indicated mutants. The *Y*-scale is logarithmic in “a” and “c,” but linear in “e.” b, d, f) The same, but for the terminus. Again, the *Y*-scale is logarithmic in “b” and “d,” but linear in “f.”

Our viability studies show that the DNA degradation activity of the RecBCD enzyme is essential for the *dnaA* mutant at the semipermissive temperature, most likely facilitating replication, but it was unclear which replication stage was more affected, initiation or elongation. To identify the most affected replication stage in the double *dnaA recBC* mutant at 39°C, we followed the time course of accumulation of the chromosomal origin signal vs the terminus signal, comparing the double mutant to the corresponding single mutants (the *recBC* mutant in this test behaves essentially as WT) (Fig. 7a and b). Thus, we compared replication initiation (by quantifying *oriC*) with replication elongation (by quantifying the *ter* region) (Rao and Kuzminov 2019), in the deep dilution conditions (Fig. 1e).

The single *recBC* mutant showed robust initiation at 39°C: the 16-fold increase in *oriC* within the first hour (Fig. 7a) indicates 4 initiation rounds. The replication elongation in the *recBC* mutant, indicated by the matching terminus increase, was equally robust (Fig. 7b). In contrast, *dnaA* and *recBC dnaA* cells show a single initiation event at 39°C, after which *dnaA recBC* stops further initiations, while *dnaA* makes another one by 2 h and hints at another “half-round” by 4 h (Fig. 7a). At the same time, existing RFs in the *dnaA* single and *dnaA recBC* double mutants are operational, although inhibited compared with *recBC*, as the increase in the terminus quantity indicates (Fig. 7b). In particular, the *dnaA recBC* mutant manages to finish 2 replication rounds by 2 h before plateauing, while the *dnaA* single mutant finishes 3 rounds by 2 h and also pauses, but seems poised to resume replication after 3 h (Fig. 7b). In general, however, the 2 mutants show little difference in their degree of initiation inhibition and slower elongation progression within the first 3 h at 39°C, suggesting that the RecBCD effects start later.

We confirmed that this broad replication inhibition is the primary defect in the *dnaA* mutant by doing the same origin and terminus copy number time course analysis in the 2 *dnaA recBC* colethality suppressors, *seqA* and *hslUV*. We observed that both suppressors significantly improved origin initiation in the mutant (Fig. 7c), while also improving replication elongation in the *seqA* mutant, but not much in the *hslUV* mutant (Fig. 7d), consistent with a better growth of the *dnaA recBC seqA* compared to the *dnaA recBC hslUV* (Fig. 2, a and c). In fact, elongation inhibition was expected in the *hslUV* suppressor, because in the absence of the HslUV degradation, the increased copy number of the DnaA46 protein should increase its overall binding to the chromosome.

In contrast with the *dnaA recBC* colethal, the *dnaN recBC* colethal shows a milder replication defect, which appears no different than in the single *dnaN* mutants (Fig. 7, e and f). In particular, the origin increase in the first 2 h is the same for the 3 strains (*recBC*, *dnaN*, and *recBC dnaN*) and only then becomes slower in the *dnaN* mutants (Fig. 7e). The terminus increase in both *dnaN* mutant strains is more modest (Fig. 7f), reflecting the expected elongation problems. Looking closer, it becomes apparent that the 2 curves (for origin and terminus) are almost superimposable for the *dnaN* single mutant (compare Fig. 7, e vs f), suggesting a modest elongation defect. In contrast, in the *dnaN recBC* mutant, the origin increase curve was twice as steep as the terminus increase one (compare Fig. 7, e vs f), indicating significant elongation problem without RF repair in the *dnaN* mutant. This finding is in agreement with the significant chromosome fragmentation in this mutant (Fig. 6, e and f). Overall, we conclude that the *dnaA recBC* mutant stops initiations and slows down elongation at the nonpermissive temperatures, while the *dnaN recBC* mutant at first initiates normally at the nonpermissive temperature, but soon runs into problems with elongation, and its initiation also becomes affected.

#### RecBCD restarts RFs frozen in the *dnaA* mutant

**Fig. 8.**
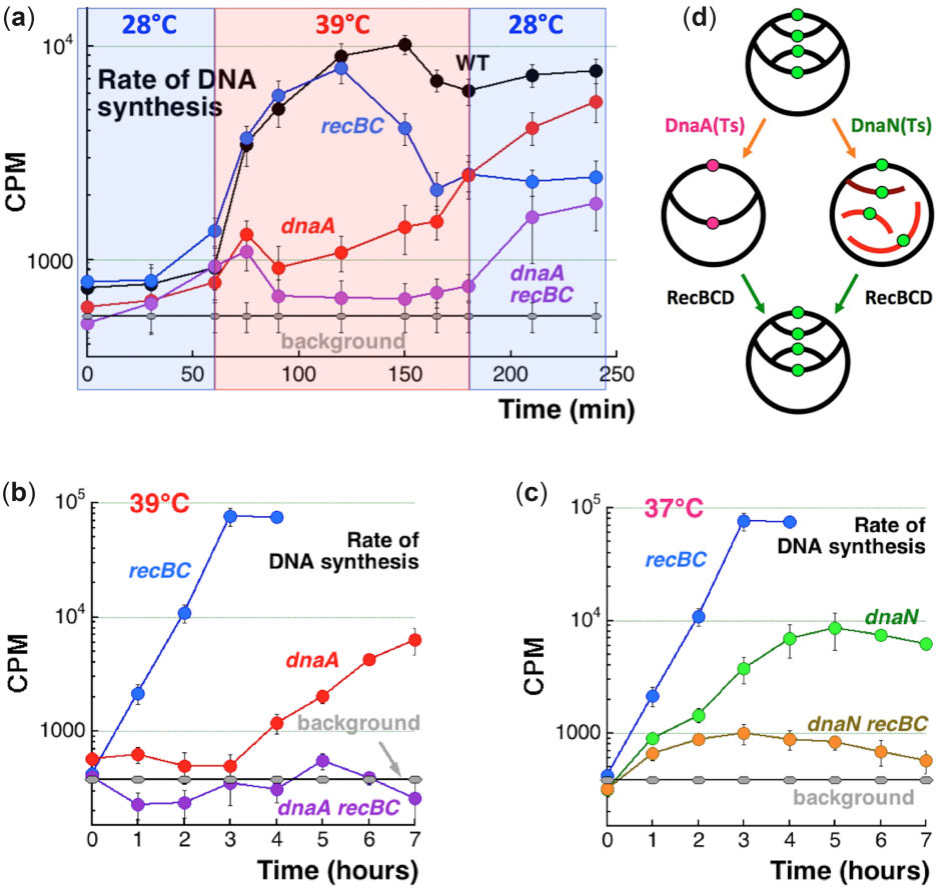
The rate of DNA synthesis. In *Y*-axis, CPM = “counts per minute.” a) The rate of DNA synthesis in the indicated strains at the indicated temperatures. At the time 60', there was a 28°C → 39°C shift-up, while at 180', there was a 39°C → 28°C shift-down. The cultures were not diluted. The average background ± SEM is shown as the gray line. b, c) The rate of DNA synthesis of the indicated mutants at the indicated semi-permissive temperatures for single *dna* mutants, in ×30 diluted cultures. d) Interpretation of the replication defects of the *dnaA* versus *dnaN* mutants and their “repair” by RecBCD.

Puzzled by the apparent lack of RecBC effect for chromosomal replication in the *dnaA* and *dnaN* mutants (Fig. 7), we measured a different replication parameter, and for longer times. To see whether RecBCD works continuously to support new initiations in the *dnaA* mutant, or reacts to initiation problems only after they arise, we used ^3^H-dT incorporation to compare the rate of DNA replication in the *dnaA* single vs *dnaA recBC* double mutant cultures at various temperatures (Fig. 8). For practical reasons, the initial experiments were done with undiluted cultures, when the viability loss of the double mutant was minimal (Fig. 1d). The 4 strains (the WT, the 2 single mutants and the double mutant) were grown at 28°C till they reached OD = 0.1, at which time (T = 0) we started measuring their DNA synthesis rate at regular intervals, while the strains were continued at 28 °C (Fig. 8a). After 60 min at 28 °C (at which point all 4 strains still showed comparable DNA synthesis rates), the 4 cultures were shifted to 39 °C for 120 min, then brought back to 28 °C for another 60 min (Fig. 8a).

The wild-type and the *recBC* single mutant showed comparable and high rates of DNA synthesis at either 39°C or later at 28°C, although half-way through 39°C the rates stabilized, indicating exit out of logarithmic growth (Fig. 8a). Also, the *recBC*(Ts) mutant significantly reduced the rate after this stabilization at 39°C, likely because of its inability to reassemble disintegrating RFs at this temperature. In contrast, the *dnaA* single and the *dnaA recBC* double mutants showed an initial brief spike after 15' at 39°C, followed by gross inhibition of DNA synthesis rates after 30' at 39°C (Fig. 8a). Thus, our DNA synthesis rate measurements supported the earlier conclusion about inhibited elongation combined with deficient initiation in the *dnaA*(Ts) mutant after shifting to 39°C. The DNA synthesis rates in the *dnaA recBC* double mutant remained flat while the strain was at 39°C, recovering only when the strain was returned to 28°C. In contrast, the *dnaA* single mutant gradually resumed replication at 39°C, revealing the action of RecBCD, but reaching WT levels only after return to 28°C (Fig. 8a).

To make sure our observations in standard-dilution cultures were robust, we also measured DNA synthesis rate in deeply diluted cultures, in which the double mutant dies (Fig. 1e), incubating them at 39°C for 7 h straight. Under these more stringent conditions, the rate of DNA synthesis in the *dnaA recBC* double mutant never recovered above the background, while the rate in the *dnaA* single mutant did eventually recover, but only after a 3-h lag (Fig. 8b), validating the earlier hint of recovery (Fig. 7, a and b). Thus, the RecBCD enzyme does not act continuously in the *dnaA*(Ts) mutant at the semi-permissive temperature, but eventually starts facilitating replication, both the initiation and elongation stages.

The situation was again quite different in the *dnaN* vs *dnaN recBC* mutant at 37°C (their semi-permissive temperature) (Fig. 8c): although both mutants kept similarly reduced levels of replication at the semi-permissive temperature for 2 h, the *dnaN* single mutant then continued with a brief exponential increase, while the *dnaN recBC* double mutant leveled off and reduced its replication, indicating elongation crisis. Thus, in the *dnaN*(Ts) mutant at 37°C, RecBCD clearly supports the integrity of RFs (Fig. 8d).

## Discussion

The *recBCD* defect in the major double-strand break repair and linear DNA degradation activity of *E. coli* is synthetic lethal with various other defects in the DNA metabolism, for example, *dut*, *rnhAB*, or *ligA*—rationalized by the increased frequency of double-strand DNA breaks in these mutants (Kouzminova and Kuzminov 2004, 2012; Kouzminova *et al.* 2017). In all the cases, massive chromosome fragmentation is readily detectable in the double mutants and requires active RFs. RFs in general are the most vulnerable chromosomal points—and RecBCD-dependence of various replication elongation mutants provides ample confirmation of this concept (Michel *et al.* 1997; Flores *et al.* 2001; Grompone *et al.* 2002). At the same time, defects in replication initiation were not expected to lead to double-strand DNA breaks—for the simple reason that they tend to align the chromosomes (making them free of RFs), by allowing existing replication rounds to complete and at the same time blocking new initiations. Therefore, our original finding that the *dnaA* and *dnaC* defects in replication initiation are synthetic lethal with the *recBC* defect (Fig. 1) appeared to make no sense. Suppressor analysis revealed an unexpected complexity, as the 2 mutants were *recBC*-colethal for opposite reasons: too much initiation in the *dnaC* mutants, while too little in the *dnaA* one. We decided to concentrate on the *dnaA* mutant defect, as the one without an obvious explanation, but with a hint at the possibility of a replication initiation role for the RecBCD enzyme.

We found that the *dnaA* mutant relies on the DNA degradation function of RecBCD, with either undetectable or a slightly poisonous role of recombinational repair (Fig. 4), but some roles for HJ migration (Fig. 5). Moreover, suppressors of the *dnaA recBC* colethality point to insufficient initiation, suggesting that there is not enough initiation-competent DnaA(Ts) protein, while linear DNA degradation somehow compensates for the defect, and HJs are involved. Therefore, we proposed that the mutant DnaA protein [known to bind DNA more readily than WT DnaA (Carr and Kaguni 1996)] has problems dissociating from its multiple chromosomal binding sites and thus stalls RFs (Fig. 5c). The stalled RF reversal, followed in RecBCD^+^ cells by linear tail degradation, resets the forks, while transient recruitment of Rep makes the reset forks powerful enough to break through the DnaA barriers (Fig. 5c). Steady progress of such “turbo” RFs allows not only to keep replication elongation going, but also to displace from the template DNA enough DnaA for initiation of new replication rounds (Fig. 5d). But this RF reset is disabled in the *recBC*(Ts) mutant at 37°C and higher, due to RecBCD inactivation.

Testing various predictions of this model yielded supporting results (Figs. 6–8), even though for physical studies we had to switch from “several day incubation on plates” time frame to “several hours in liquid cultures” time frame. In particular, it was found that the *dnaA recBC* mutants do not fragment their chromosome (Fig. 6, e and f) and that replication elongation and initiation are both completely blocked in the mutant (Figs. 7 and 8). We conclude that linear DNA degradation by RecBCD compensates for the chromosomal defects of the partially active DnaA mutant protein by resetting and thus “invigorating” the inhibited RFs.

The proposed tighter DNA binding by the mutant DnaA in vivo appears to be directly testable by whole-cell methods like ChIP-seq or Gfp-DnaA imaging. However, further examination of this idea makes the differences hard to detect, since the mutant protein is expected to bind the same chromosomal sites and at the same general copy number as the WT DnaA. The only proposed difference is the rate of dissociation upon RF arrival, which would be hard to see by methods looking at the entire chromosome. In the absence of obvious tests of this idea in vivo, we have to broaden possible causative defects of the DnaA(Ts) protein to include such formal alternatives as decreased nucleotide binding at higher temperature, decreased ATP-dependent filament formation at *oriC* or increased protein aggregation.

### Oddities

In this section we briefly touch on several unusual observations without obvious explanations. The density-dependent survival of the double mutant (Fig. 1, d and e) could be easily explained if related to cellular scavenging systems, like catalases, which do offer better protection at higher cell densities—but nothing of this kind is known for either DnaA or RecBCD mutants. In general, density-dependent survival probably reflects the presence of various components of the quorum sensing systems in *E. coli* (Ahmer 2004).

Although RuvAB and RecG are analogs in that they both migrate Holiday junctions along the DNA molecules (Lloyd and Sharples 1993; Eggleston *et al.* 1997), and the corresponding mutants both decrease homologous recombination or survival after DNA damage (Lloyd 1991), the 2 mutants have the opposite phenotypes in our genetic system: *ruvAB* inhibits *dnaA*(Ts) growth at the semi-permissive temperature, while *recG* enhances it (Fig. 5, a and b). There are only a few reports of the *ruvAB* and *recG* defects acting in opposite directions, and in all of them the *ruvAB* defect lowers the WT numbers, while the *recG* defect increases them. One such genetic system is deletion between tandem repeats in plasmids (Lovett *et al.* 1993), another is recombinational repair of a chromosomal double-strand cut off a partially homologous plasmid (Grove *et al.* 2008), and perhaps the best known one is the “adaptive mutagenesis” (Foster *et al.* 1996; Harris *et al.* 1996). The disparate effects of the 2 defects in HJ-moving enzymes, as well as the difference in their genetic suppressors, support the idea that their cellular functions, once postulated for both to be processing of recombinational repair intermediates (Lloyd 1991; Lloyd and Sharples 1993), are actually quite different: removal of HJs for RuvABC, while preventing over-replication after recombinational repair for RecG (Zhang *et al.* 2010). It is, in fact, this “overreplication,” which allows the *recG* defect to suppress the *dnaA46* defect at the nonpermissive temperature of 42°C, in the special Δtus rpoB*35 background (Rudolph *et al.* 2013).

Dependence of a particular mutant on RecBCD does not always mean that the mutant is equally dependent on recombinational repair (RecA). Although similar RecA- and RecBCD-dependence is typical (Kouzminova *et al.* 2004), there are mutants, like *rep* (auxiliary replicative helicase) or *rnhA* (RNase HI), that depend more on linear DNA degradation by RecBCD and less on recombinational repair—these mutants can survive *recA* inactivation (even though they are usually inhibited by RecA loss) (Itaya and Crouch 1991; Uzest *et al.* 1995). At the same time, the *dnaA* mutant is the first RecBCD-dependent mutant (as far as we know) that shows complete lack of fragmentation in the *dnaA recBC* format—this makes its RecBCD-dependence even more perplexing. To clarify, the absence of chromosomal fragmentation in the *dnaA recBC* mutants does not mean the absence of dsDNA ends [the only known substrates of RecBCD (Dillingham and Kowalczykowski 2008)]—only that the likely formation of double-strand ends in the chromosome of *dnaA recBC* mutants does not release subchromosomal fragments [like it does for *dnaN recBC* mutant (Fig. 8d)]. Perhaps in this mutant, open DNA ends are parts of bigger chromosomal structures, like regressed RFs or sigma-replicating chromosomes (Fig. 5c), which do not yield linear subchromosomal pieces in pulsed-field gels. Mechanistically, with the demonstration (Figs. 7 and 8) that replication is inhibited in the mutant indicating that RFs are stalled, the lack of chromosome fragmentation means that the RuvC resolvase does not have access to the regressed RFs, even though RuvAB apparently moves them around.

Another unusual finding is the effect of *datA* copy number changes in the *dnaA recBC* mutant. The natural expectation is, if a deletion of a genetic element (a gene or a locus) changes a phenotype one way, then an increased copy number of this genetic element should change the phenotype in the opposite direction. In the case of *dnaA recBC* mutant, both *datA* deletion and additional plasmid-borne copies of *datA* exacerbate the colethality phenotype (Fig. 6, a and b). Although we rationalized the same direction of both effects, the situation is still an unusual one. In fact, the only other similar situation that we are aware of is the influence of the *dam* gene copy number on mutagenesis. In the *dam* mutants, mutagenesis is increased because of the disoriented mismatch repair (Marinus and Morris 1974). Unexpectedly, mutagenesis is also increased by Dam+ overproduction—because now mismatch repair has a reduced window of opportunity due to faster methylation of the nascent DNA (Marinus *et al.* 1984). In the *dam* gene situation, the formal genetic analysis has to yield to the advanced mechanistic understanding of the mismatch repair phenomenon. Hopefully the mechanistic assumptions in the basis of our analysis are as solid.

### The complex nature of synthetic lethality

The phenomenon of synthetic lethality (colethality) (Guarente 1993; Nijman 2011), frequently comes as a surprise, although it should be expected. Logically, if there is an essential function, inactivation of which leads to cell inviability [and there are hundreds of essential functions (Gerdes *et al.* 2003)], there could be 2 different enzymes in the same cell performing this same function (Ting *et al.* 2008), providing the level of “buffering via redundancy” the biological systems are famous for (Tautz 1992; Wilkins 1997; Hartman *et al.* 2001). In fact, this natural explanation of synthetic lethality as a combination of 2 mutations inactivating the 2 alternative branches of the same essential pathway is still the most intuitive one (Guarente 1993; Hartman *et al.* 2001; Ooi *et al.* 2006). However, as argued before, the majority of synthetic lethal combinations are avoidance-repair couples, in which one defect allows a potentially lethal problem to arise frequently, while the other defect blocks the repair of this problem (Ting *et al.* 2008; Mahaseth and Kuzminov 2017).

The lethality of *dnaA recBC* combination cannot be due to redundancy for obvious reasons, but is it the “avoidance-repair couple” type? In such a couple, RecBCD would be, naturally, the repair activity, while the DnaA would have to be the avoidance activity. But what DNA lesion is repaired or avoided in this case? If the repair enzyme is RecBCD, its only known substrates are double-strand DNA breaks (Dillingham and Kowalczykowski 2008), and these are not detected in the double mutant (Fig. 6, e and f). What is clear from our physical analysis is that RecBCD acts to help DnaA mutant protein to perform its activity—the initiation of chromosomal replication at *oriC*. From this perspective, the *dnaA recBC* colethality looks like another case of “compensation,” when a hypomorph mutant of an essential function is operational as long as it is supported by other cellular activities; when this support is withdrawn by additional mutations, the hypomorph fails in its function. For example, the temperature-sensitivity of *dnaA46* mutant is suppressed in *pta* and *ackA* mutants that accumulate acetate and pyruvate (Tymecka-Mulik *et al.* 2017), suggesting mutant protein compensation by cytoplasmic chemistry.

The idea of compensation is analogous to the concept of “biochemical buffers” explaining how HSP90 chaperone activity essentially hides the genetic diversity of tumors, canalizing their phenotypes (Whitesell and Lindquist 2005). In fact, overproduction of GroEL/GroES, the major protein chaperone in *E. coli*, relieves temperature-sensitivity of the *dnaA46*(Ts) mutant (Fayet *et al.* 1986), demonstrating compensation in action. Obviously, compensation as a colethality phenomenon is possible only when one of the 2 colethal mutations is a hypomorph—and both the *dnaA*(Ts) and the *recBC*(Ts) defects in this case fulfill this requirement. We have encountered cases of compensation colethality before with the *dut-1* defect, also a hypomorph of an essential gene (Ting *et al.* 2008). However, the *dnaA recBC* case cannot be compensation only, because the *dnaA* defect does lead to a chromosomal problem (inhibited replication, HJs), which, in the form of regressed RFs, is “repaired” by RecBCD-promoted degradation—thus, indirectly arguing for the avoidance-repair couple relationship. We propose that *dnaA recBC* is a hybrid case of synthetic lethality that has elements of both the avoidance-repair couple and compensation.

### Conclusion: the DnaA-chromosome balance

RecBCD, as the most powerful helicase/nuclease of bacterial cells, was always considered a major supporting function for replication *elongation*, ensuring the steady progress of RFs via linear DNA degradation at regressed forks or via RecA-catalyzed recombinational repair of disintegrated forks (Kuzminov 1999; Dillingham and Kowalczykowski 2008). Here, we describe the situation, in which RecBCD definitely supports replication *initiation*, even though indirectly, by performing its main function, ensuring progress of RFs. In other words, the steady RF progress is important for the timely initiation in the *dnaA* mutant, reflecting the complex interplay between elongation and the regulation of new initiation rounds, achieved via the DnaA protein binding to, and displacement from, the chromosomal DNA. DnaA binds to more than 300 sites throughout the chromosome (Hansen and Atlung 2018), but can polymerize only at *oriC* and a few other sites (Roth and Messer 1998). The balance between the general chromosome binding vs *oriC* polymerization is apparently upset in our *dnaA*(Ts) mutants in favor of the chromosome, making steady RF progress a prerequisite for rebalancing. Overproduction of some DnaA(Ts) proteins were reported to inhibit replication elongation at lower temperatures (Nyborg *et al.* 2000). Thus, *dnaA recBC* colethality highlights the chromosomal aspect of the regulation of replication initiation. There is also the cell growth aspect, pertaining to the copy number of DnaA per cell, which was not monitored in our study.

## Supplementary Material

jkac228_Supplementary_Data

## Data Availability

Strains and plasmids are available upon request. The authors affirm that all data necessary for confirming the conclusions of the article are present within the article, figures, and tables. Supplemental material is available at G3 online.
