# Supplementary material for: Robust linear DNA degradation supports replication–initiation-defective mutants in Escherichia coli
Source: G3 (Bethesda). 2022 Sep 27;12(11):jkac228. doi: 10.1093/g3journal/jkac228 (PMC9635670; doi:10.1093/g3journal/jkac228)
Supplement: jkac228_Supplementary_Data [file jkac228_supplementary_data.pdf]

Supplement

For the paper by

T.V. Pritha Rao* and Andrei Kuzminov

Robust linear DNA degradation supports replication-initiation-defective mutants in *Escherichia coli*

Characterization of the *dnaK* suppressor of the *dnaA recBC* colethality

We have isolated a single suppressor upstream of *dnaK*. The DnaK chaperone (together with GrpE) is known to relieve DnaA46(Ts) protein thermolability in vitro (CARR AND KAGUNI 1996). Since the insertion in the *dnaK* gene was upstream of the promoter (Fig. S1A), we tried both overexpression of DnaK and DnaJ proteins, as well as deletion of the *dnaK* gene, to reveal the nature of a possible *dnaK* suppression. Overexpression of the DnaK, or both DnaK and DnaJ, from the plasmids pNRK416 and pMob-*dnaKJ* (BREDÈCHE *et al.* 2001), did not rescue the *dnaA recBC* strain at 39°C, while being inhibitory for the single *dnaA* mutant at 39°C (Fig. S1C). Unfortunately, we have not been able to create a Δ*dnaK recBC dnaA* by transduction. We have also found no difference in the viability of *dnaA recBC* strain at 39°C upon overexpression of *yaaI* and *yaaW,* the two genes upstream of *dnaK* (not shown).

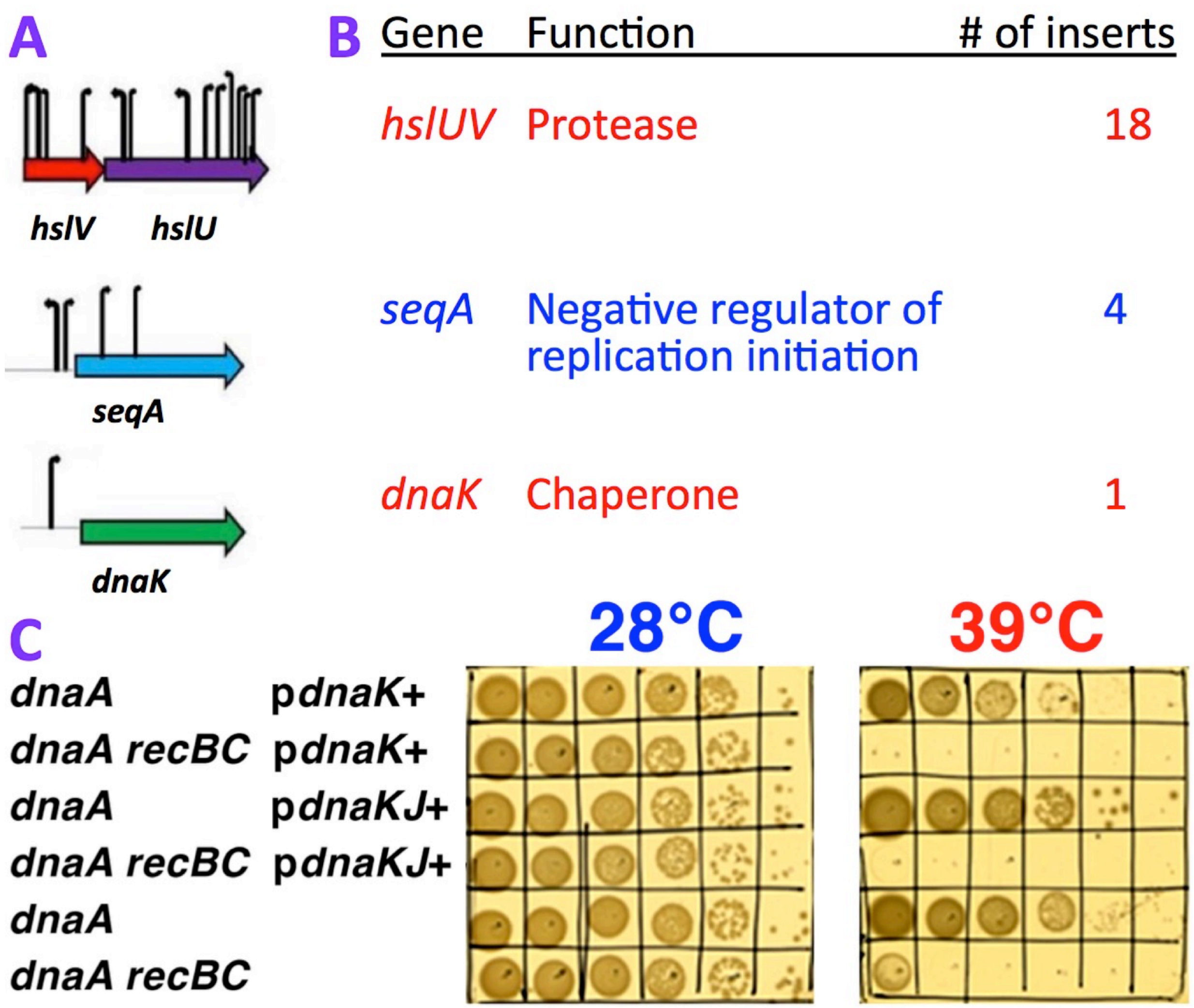

Fig. S1. The *dnaA recBC* colethality suppressors.

A. Position of inserts.

B. Statistics and functions of the inactivated genes. Color-coding: blue, DNA metabolism; red, protein degradation or chaperone.

C. Overexpression of either *dnaK*+ alone or *dnaKJ*+ together exacerbates the *dnaA recBC* colethality at 39°C.

A

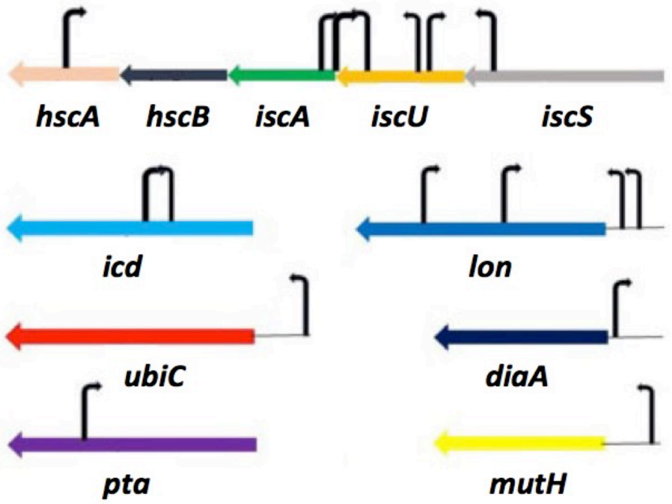

B

Gene	Function	# of inserts
iscSUA hscA	Fe-S cluster assembly	7
lon	Protease	4
icd	isocitrate dehydrogenase	2
ubiC	chorismate lyase (biosynthesis of ubiquinone)	1
pta	phosphate acetyltransferase	1
diaA	ATP-DnaA polymerization at *oriC*	1
mutH	Mismatch repair	1

Fig. S2. The *dnaN recBC* suppressors.

A. Position of inserts.

B. Statistics and functions of the inactivated genes. Color-coding: blue, DNA metabolism; green, central metabolism; red, protein degradation.

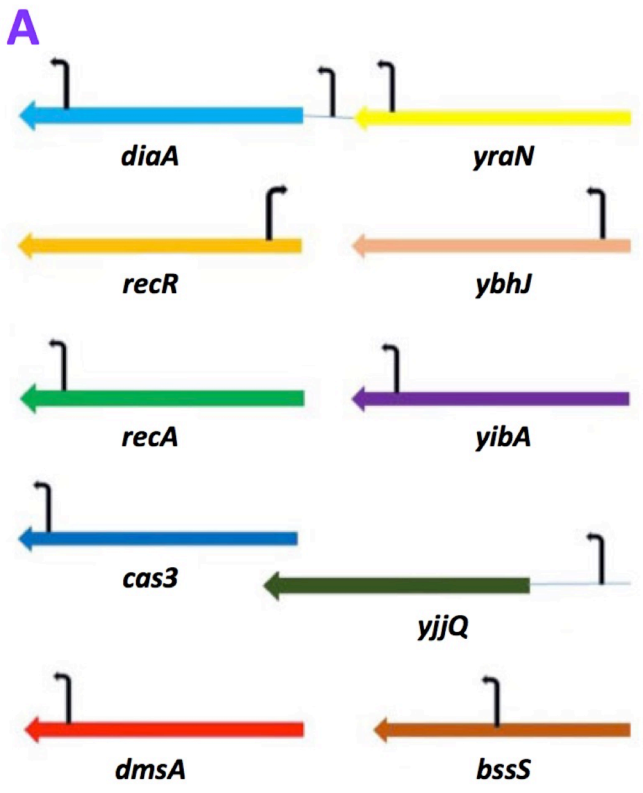

B

Gene	Function	# of inserts
diaA	ATP-DnaA polymerization at *oriC*	2(3)
recA	Recombinational repair	1
recR	Recombinational repair	1
cas3(ygcB)	CRISPR helicase/nuclease	1
dmsA	dimethyl sulfoxide reductase	1
bssS	Regulator of biofilm formation (pleiotropic)	1
yibA	Unknown (stress tolerance)	1
ybhJ	Unknown (oxidative stress?)	1
yjjQ	Unknown	1

Fig. S3. The *dnaC recBC* suppressors.

A. Position of inserts.

B. Statistics and functions of the inactivated genes. Color-coding: blue, DNA metabolism; green, central metabolism.

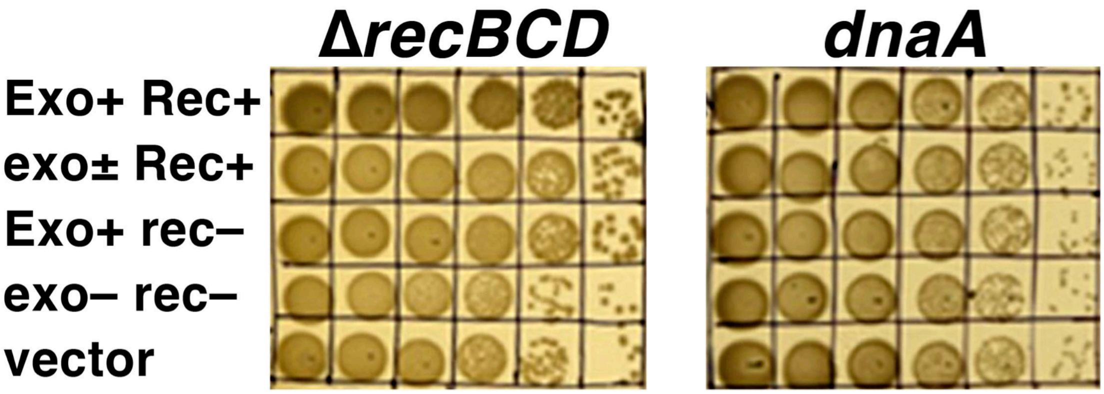

Fig. S4. Controls for the plasmids carrying various *recBCD* alleles: plating in the Δ*recBCD* (JB1) versus *dnaA* (SRK309-1) single mutants at 39°C.

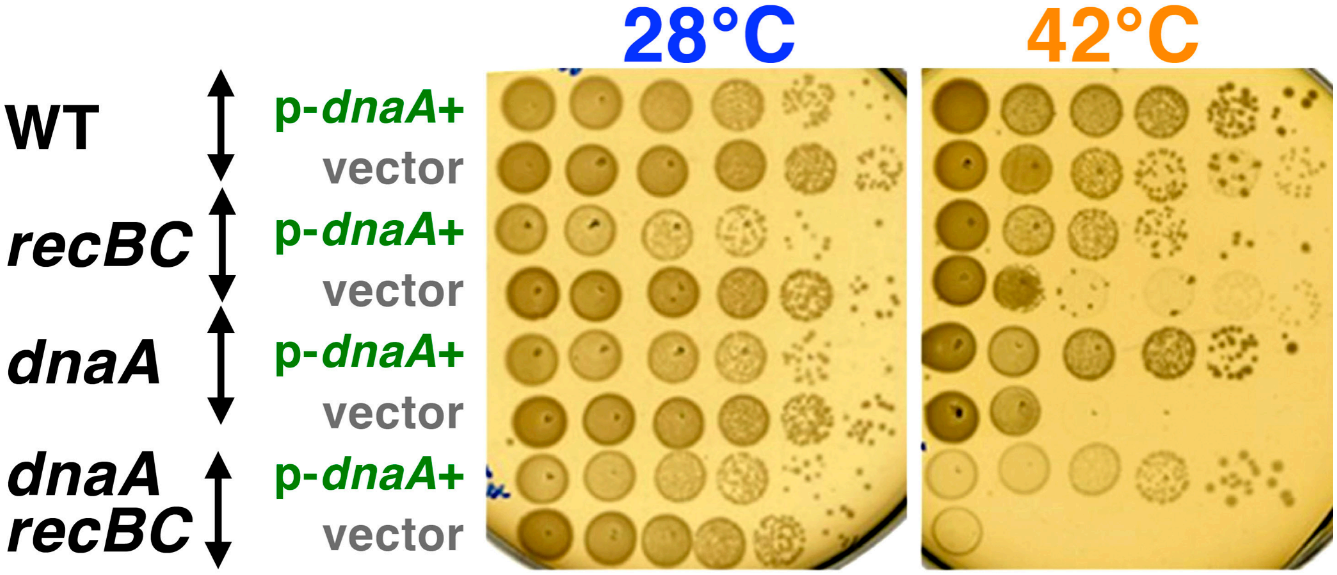

Fig. S5. The effect of high copy number WT DnaA in the same strains as in Fig. 6C. Note the vector-alone inhibition at 42°C.

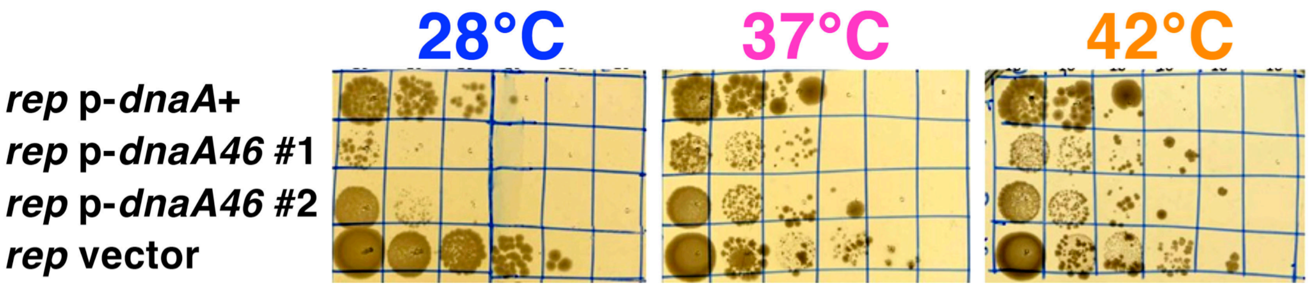

Fig. S6. The effects of overproduction of DnaA+ and DnaA46 proteins in the *rep* mutant. Even vector-alone shows some inhibition at 37°C and 42°C.

Table S1. Strains used.

Strain	Genotype	Reference/Source
**Published**		
AB1157	Wild type	(BACHMANN 1987)
BT125	*recD1011*	(RINKEN *et al.* 1992)
JB1	Δ*recBCD3*::*kan*	(MIRANDA AND KUZMINOV 2003)
JC7623	*recB21 recC22 sbcB15 sbcC201*	(KUSHNER *et al.* 1971)
SK129	*recB270 recC271*(Ts)	(KUSHNER 1974)
SRK309-1	AB1157 *dnaA46*(Ts)	(KHAN AND KUZMINOV 2019)
SRK309	AB1157 *dnaA46*(Ts) *tna::Tn10 recBC*(Ts)	SK129 x P1 NS373
HC123	*dnaN159*(Ts)	(SAKAKIBARA AND MIZUKAMI 1980)
L392	AB1157 *dnaC2*(Ts)	(KOUZMINOVA AND KUZMINOV 2012)
GY9701	*recA938::cam* miniF-kan *recA*+	Raymond Devoret via Benedicte Michel
CM2500	*dnaA601*(Ts)	(HANSEN *et al.* 1992)
JW1850-2	Δ*ruvA786*::*kan*	(BABA *et al.* 2006)
JJC213	Δ*rep*::*kan*	(UZEST *et al.* 1995)
JW3118-2	Δ*diaA762*::*kan*	(BABA *et al.* 2006)
N2731	*recG258::mini*-Tn*10 kan*	(LLOYD AND BUCKMAN 1991)
NS373	*dnaA46(Ts) tna::Tn10*	(SCHAUS *et al.* 1981)

**This study**		
RA69	*dnaN159*(Ts) *yidX+ kan yidA+*	Precise insertion in HC123
RA70	AB1157 *dnaN159*(Ts) *yidX+ kan yidA+*	AB1157 x P1 RA69
RA71	AB1157 *dnaN159*(Ts) (*kan*) *recBC*(Ts)	SK129 x P1 RA69
RA72	AB1157 *dnaC2*(Ts) *recBC*(Ts)	SK129 x P1 L392
RA73	AB1157 *dnaA46*(Ts) *recBC*(Ts) *hslUV*::pRL27	SRK309 transformed with pRL27
RA74	AB1157 *dnaA46*(Ts) *recBC*(Ts) *seqA*::pRL27	SRK309 transformed with pRL27
RA75	AB1157 *dnaA46*(Ts) Δ*recBCD3::kan*	SRK309-1 x P1 JB1
RA76	AB1157 *dnaA46*(Ts) *recA938::cat*	SRK309-1 x P1 GY9701
RA77	AB1157 *dnaA601*(Ts)	AB1157 x P1 CM2500
RA78	AB1157 *dnaA601*(Ts) *recBC*(Ts)	SK129 x P1 CM2500
RA79	AB1157 *dnaA601*(Ts) *recBC*(Ts) *hslUV*::pRL27	RA78 x P1 RA73
RA80	AB1157 *dnaA601*(Ts) *recBC*(Ts) *seqA*::pRL27	RA78 X P1 RA74
RA81	AB1157 *dnaA46*(Ts) *ruvA786::kan*	SRK309-1 x P1JW1850-2
RA82	AB1157 *dnaA46*(Ts) *recG258::mini*-Tn*10 kan*	SRK309-1 x P1 N2731
RA83	AB1157 *dnaA46*(Ts)	RA81 treated with pCP20 x

	ruvA786 recG258	P1N2731
RA84	AB1157 *dnaA46*(Ts) *recD1011*	BT125 x P1 NS373
RA85	AB1157 *rep recBC*(Ts)	SK219 x P1 JJC213
RA86	AB1157 *rep dnaA46*(Ts)	SRK309-1 x P1 JJC213
RA87	AB1157 *rep dnaA46*(Ts) *recBC*(Ts)	RA85 x P1 NS373
RA88	AB1157 *dnaN159*(Ts) *recBC*(Ts) *icd*::pRL27	RA71 transformed with pRL27
RA89	AB1157 *dnaN159*(Ts) *recBC*(Ts) *lon*::pRL27	RA71 transformed with pRL27
RA90	AB1157 *dnaN159*(Ts) *recBC*(Ts) *iscU*::pRL27	RA71 transformed with pRL27
RA91	AB1157 *dnaN159*(Ts) *recBC*(Ts)	RA71 treated with pCP20
RA92	AB1157 *dnaN159*(Ts) *recBC*(Ts) Δ*diaA::kan*	RA91 X P1 JW3118-2
RA93	AB1157 *dnaN159*(Ts) *recBC*(Ts) *ruvA786::kan*	RA91 x P1 JW1850-2
RA94	dnaN159 recA938	RA70 x P1 GY9701
RA95	AB1157 *dnaC2*(Ts) *recBC*(Ts) *recA*::pRL27	RA72 transformed with pRL27
RA96	AB1157 *dnaC2*(Ts) *recBC*(Ts) *bssS*::pRL27	RA72 transformed with pRL27
RA99	AB1157 *dnaC2*(Ts) *recBC*(Ts) Δ*diaA::kan*	RA72 x P1JW3118-2
RA99	AB1157 *dnaA46*(Ts) *recBC*(Ts) *recR*::pRL27	RA72 transformed with pRL27
RA100	AB1157 *dnaA46*(Ts) *recBC*(Ts) *diaA*::pRL27	SRK309 x P1 RA99

RA102	AB1157 *dnaA46*(Ts) *recBC*(Ts) *diaA*::pRL27	SRK309 x P1 RA99
RA103	AB1157 Δ*datA*::kan	Precise deletion of *datA*
RA104	AB1157 *recBC*(Ts) Δ*datA*::kan	SK129 x P1 RA103
RA105	AB1157 *dnaA46*(Ts) Δ*datA*::kan	SRK309-1 x P1 RA103
RA106	AB1157 *dnaA46*(Ts) *recBC*(Ts) Δ*datA*::kan	SRK309 x P1 RA103
RA107	AB1157 *dnaA46*(Ts) *recBC*(Ts) *recA938::cat*	SRK309 x P1 GY9701
RA108	AB1157 *dnaA46*(Ts) *recBC*(Ts) *ruvA786*	SRK309 x P1JW1850-2
RA109	AB1157 *dnaA46*(Ts) *recBC*(Ts) *dnaK*::pRL27	SRK309 transformed with pRL27
RA130	AB1157 *dnaA46*(Ts) *recB21 recC22 sbcB15 sbcC201*	JC7623 x P1 SRK309-1
RA131	AB1157 *dnaA601*(Ts) *seqA*::pRL27	RA77 x P1 RA74
RA132	AB1157 *dnaA601*(Ts) *hslUV*::pRL27	RA78 x P1 RA73

Table S2. Plasmids used.

Name	Replicon / drug resistance / relevant genes	Reference/Source
pCP20	Rep^{ts} / *bla cat* / *flp*	(CHEREPANOV AND WACKERNAGEL 1995)
pMTL20	ColE1 high copy #/ *bla* / *lacZ alpha*	(CHAMBERS *et al.* 1988)

pACYC177	P15A/ *bla kan*	(CHANG AND COHEN 1978)
pKD46	Rep^{ts}/ *bla* / *exo gam bet araC*	(DATSENKO AND WANNER 2000)
pSA122	p15A / *cat* / *recB-recC*	(AMUNDSEN *et al.* 2000)
pAMP1	pSC101/ *bla/ recC-ptr-recB-recD*	(MIRANDA AND KUZMINOV 2003)
pAMP3	pSC101/ *bla* / *recC-ptr-recB*	(MIRANDA AND KUZMINOV 2003)
pAMP5	pSC101/ *bla* / *recC-ptr-recB*-recD*	(MIRANDA AND KUZMINOV 2003)
pAMP7	pSC101/ *bla* / *recC-ptr-*$recB^{1080}$*-recD*	(MIRANDA AND KUZMINOV 2003)
pWSK29	pSC101/ *bla*	(WANG AND KUSHNER 1991)
pNRK416	/ *bla* / *dnaK*	(BREDÈCHE *et al.* 2001)
pMob45	pMob/ *bla* / *dnaK dnaJ*	(BREDÈCHE *et al.* 2001)
pPR1	pMTL20 / *bla* / *lacZ alpha dnaA-dnaN*	This study
pPR2	pMTL20 / *bla* / *lacZ alpha dnaN-dnaA*	This study
pPR7	pMTL20 / *bla* / *lacZ alpha dnaA*	This study
pPR8	pMTL20 / *bla* / *lacZ alpha dnaN*	This study
pPR9	pMTL20 / *bla* / *dnaA*	This study
pPR10	pMTL20 / *bla* / *dnaA46*(Ts)	This study
pPR11	pMTL20 / *bla* / hslUV	This study
pMOR6	P15A/ *bla kan* /*datA*	(MORIGEN *et al.* 2001)

References

Amundsen, S. K., A. F. Taylor and S. G.R., 2000 The RecD subunit of the *Escherichia coli* RecBCD enzyme inhibits RecA loading, homologous recombination, and DNA repair. Proc. Natl. Acad. Sci. USA 97**:** 7399-7404.

Baba, T., T. Ara, M. Hasegawa, Y. Takai, Y. Okumura *et al.*, 2006 Construction of *Escherichia coli* K-12 in-frame, single-gene knockout mutants: the Keio collection. Mol. Syst. Biol. 2**:** 2006.0008.

Bachmann, B. J., 1987 Derivations and genotypes of some mutant derivatives of *Escherichia coli* K-12, pp. 1190-1219 in *Escherichia coli and Salmonella typhimurium. Cellular and Molecular Biology*, edited by F. C. Neidhardt. American Society for Microbiology, Washington, D.C.

Bredèche, M. F., S. D. Ehrlich and B. Michel, 2001 Viability of *rep recA* mutants depends on their capacity to cope with spontaneous oxidative damage and on the DnaK chaperone protein J. Bacteriol. 183**:** 2165-2171.

Carr, K. M., and J. M. Kaguni, 1996 The A184V missense mutation of the *dnaA5* and *dnaA46* alleles confers a defect in ATP binding and thermolability in initiation of *Escherichia coli* DNA replication Mol. Microbiol. 20**:** 1307-1318.

Chambers, S. P., S. E. Prior, D. A. Barstow and N. P. Minton, 1988 The pMTL nic$^-$ cloning vectors. I. Improved pUC polylinker regions to facilitate the use of sonicated DNA for nucleotide sequencing. Gene 68**:** 139-149.

Chang, A. C. Y., and S. N. Cohen, 1978 Construction and characterization of amplifiable multicopy DNA cloning vehicles derived from the p15A cryptic miniplasmid. J. Bacteriol. 134**:** 1141-1156.

Cherepanov, P. P., and W. Wackernagel, 1995 Gene disruption in *Escherichia coli*: TcR and KmR cassettes with the option of Flp-catalyzed excision of the antibiotic-resistance determinant. Gene 158**:** 9-14.

Datsenko, K. A., and B. L. Wanner, 2000 One-step inactivation of chromosomal genes in *Escherichia coli* K-12 using PCR products. Proc. Natl. Acad. Sci. USA 97**:** 6640-6645.

Hansen, F. G., S. Koefoed and T. Atlung, 1992 Cloning and nucleotide sequence determination of twelve mutant *dnaA* genes of *Escherichia coli*. Mol. Gen. Genet. 234**:** 14-21.

Khan, S. R., and A. Kuzminov, 2019 Thymineless Death in *Escherichia coli* Is Unaffected by Chromosomal Replication Complexity. J. Bacteriol. 201**:** e00797-00718.

Kouzminova, E. A., and A. Kuzminov, 2012 Chromosome demise in the wake of ligase-deficient replication. Mol. Micorbiol. 84**:** 1079-1096.

Kushner, S. R., 1974 In vivo studies of temperature-sensitive *recB* and *recC* mutants. J. Bacteriol. 120**:** 1213-1218.

Kushner, S. R., H. Nagaishi, A. Templin and A. J. Clark, 1971 Genetic recombination in *Escherichia coli*: the role of Exonuclease I. Proc. Natl. Acad. Sci. USA 68**:** 824-827.

Lloyd, R. G., and C. Buckman, 1991 Genetic analysis of the *recG* locus of *Escherichia coli* K-12 and of its role in recombination and DNA repair. J. Bacteriol. 173**:** 1004-1011.

Miranda, A., and A. Kuzminov, 2003 Chromosomal lesion suppression and removal in *Escherichia coli* via linear DNA degradation. Genetics 163**:** 1255-1271.

Morigen, E. Boye, K. Skarstad and A. Løbner-Olesen, 2001 Regulation of chromosomal replication by DnaA protein availability in *Escherichia coli*: effects of the *datA* region. Biochim. Biophys. Acta 1521**:** 73-80.

Rinken, R., B. Thoms and W. Wackernagel, 1992 Evidence that *recBC*-dependent degradation of duplex DNA in *Escherichia coli recD* mutants involves DNA unwinding. J. Bacteriol. 174**:** 5424-5429.

Sakakibara, Y., and T. Mizukami, 1980 A temperature-sensitive *Escherichia coli* mutant defective in DNA replication: *dnaN*, a new gene adjacent to the *dnaA* gene Mol. Gen. Genet. 178**:** 541-553.

Schaus, N., K. O'Day, W. Peters and A. Wright, 1981 Isolation and characterization of amber mutations in gene *dnaA* of *Escherichia coli* K-12. J. Bacteriol. 145**:** 904-913.

Uzest, M., S. D. Ehrlich and B. Michel, 1995 Lethality of *rep recB* and *rep recC* double mutants of *Escherichia coli*. Mol. Microbiol. 17**:** 1177-1188.

Wang, R. F., and S. R. Kushner, 1991 Construction of versatile low-copy-number vectors for cloning, sequencing and gene expression in *Escherichia coli*. Gene 100**:** 195-199.
